# FGFR inhibitor resistance in cervical cancer: a role for integrin α2 and mTOR signalling

**DOI:** 10.3389/fcell.2026.1863679

**Published:** 2026-06-04

**Authors:** Nauf Bou Antoun, Richard P. Grose, Anthony J. Walker, Helmout Modjtahedi, Athina Myrto Chioni

**Affiliations:** 1 School of Life Sciences Pharmacy and Chemistry, Faculty of Health, Science, Social Care and Education, Kingston University London, Kingston-upon-Thames, United Kingdom; 2 Centre for Tumour Biology, Barts Cancer Institute, Queen Mary University of London, London, United Kingdom; 3 University of Liverpool Bengaluru, Bengaluru, India

**Keywords:** cervical cancer, drug resistance, FAK, FGF(R), integrin α2, mTOR, PHLDA1

## Abstract

Cervical cancer is the fourth most common cancer among women worldwide and is often diagnosed at advanced stages, highlighting the need for effective systemic therapies, including those targeting key cancer-related pathways. Fibroblast growth factor (FGF) signalling plays a critical role in cell biology, activating FGF receptors (FGFRs) to regulate proliferation, migration and apoptosis. Aberrant activation of the pathway contributes to tumour progression in many cancers, including cervical cancer. FGFR inhibitors (FGFRi) have shown clinical promise but are limited by emergence of therapeutic resistance. Here, we describe mechanisms underpinning FGFRi resistance in three human cervical cancer cell lines (CaSki, HeLa, and SiHa). Transcriptomic analysis identified several genes differentially expressed between parental and resistant lines, including downregulation of Pleckstrin Homology Like Domain Family A Member 1 (*PHLDA1)* and upregulation of Phospholipase C beta 4 (*PLCB4)*, changes that associate with a metastatic phenotype. Protein interaction network analysis revealed integrin α2 (ITGA2) as a putative central node. Increased cytoplasmic localisation of ITGA2 and concomitant loss at cell-cell contacts, alongside focal adhesion kinase (FAK) activation was observed in PD173074 resistant cells. Moreover, resistant cell lines exhibited elevated S6 ribosomal protein (p-S6) phosphorylation, which persisted despite inhibition of FGFR, FAK or mammalian target of rapamycin complex 1 (mTORC1). Collectively, these findings suggest that FGFR inhibitor resistance in cervical cancer is associated with ITGA2–FAK signalling and AKT-independent mTOR activation, offering potential for overcoming therapeutic resistance via combinatorial treatment strategies.

## 1 Introduction

Cervical cancer remains the fourth most common cancer among woman worldwide with ∼660,000 new cases and 350,000 deaths in 2022 (WHO) (Li Z. et al., 2025). The primary cause of cervical cancer is persistent infection with high-risk human papillomavirus (HPV) types. While prophylactic HPV vaccination and regular screening can prevent cervical cancer, cultural and societal barriers, as well as limited access to prevention and screening in low- and middle-income countries hamper efficacy (Aleezada et al., 2025). Even with screening programs, cervical cancer is often diagnosed at advanced stages when symptoms occur coinciding with progression to a stage requiring systemic treatment rather than local intervention (Tekalign and Teshome, 2022).

Advanced-stage cervical cancer typically requires chemotherapy and targeted therapies. However, tumours at later stages are more genetically diverse and may have evolved intrinsic resistance mechanisms to small molecule drugs such as FGFRis (Burmeister et al., 2022). This is exacerbated by the tumour microenvironment and cellular plasticity, both of which are more pronounced in later-stage disease and contribute to therapeutic failure (Shi et al., 2023; Zaarour et al., 2023). Thus, even with targeted therapeutics, resistance (both intrinsic and acquired) is a significant barrier. In general, tumour cells may activate compensatory signalling pathways after drug treatment (e.g., Phosphoinositol-3-kinase/protein kinase B (PI3K/AKT)/mammalian target of rapamycin (mTOR), integrin/focal adhesion kinase (FAK)), undergo transcriptional reprogramming, or alter receptor expression, all of which reduce drug efficacy (Shi et al., 2023; Li et al., 2024; Aakriti et al., 2025). More specifically, resistance to a wide variety of treatments is often linked to canonical pathways like PI3K/AKT/mTOR, with downstream effectors, including phosphorylation of S6 ribosomal protein (phospho-S6), acting as crucial indicators of signalling activity (Dong et al., 2021; Yi et al., 2021). Tumour growth and treatment failure have also been linked to non-canonical pathways, such as those involving FAK and integrin signalling (Sulzmaier et al., 2014; Cifuentes et al., 2025). Such challenges underscore the need for better molecular characterization and more adaptable treatment strategies, particularly in patients with late-stage disease.

The FGFR signalling pathway is integral to various cellular processes, including proliferation, differentiation and survival (Chioni and Grose, 2021). Aberrations in FGFR signalling, such as mutations, amplifications or fusions, have been implicated in the development and progression of multiple cancers, including cervical cancer (Chioni and Grose, 2021; Mahmood et al., 2022; Bou Antoun and Chioni, 2023). Thus, FGFR inhibitors have emerged as potential therapeutic agents in cancers with FGFR alterations (Krook et al., 2021; Facchinetti et al., 2024; Zhang et al., 2024). However, the clinical efficacy of these inhibitors is often limited by the development of resistance mechanisms, which can involve activation of bypass signalling or down-stream pathways such as PI3K/AKT or Ras/mitogen-activated protein kinase (MAPK), or genetic mutations (e.g., gatekeeper mutations that hinder drug binding) and phenotypic changes like epithelial-mesenchymal transition (EMT) (Grygielewicz et al., 2016; Ryan et al., 2019; Pacini et al., 2021; Yue et al., 2021; Facchinetti et al., 2024; Zhang et al., 2024).

Previously, we demonstrated that activation of FGFR signalling in cervical cancer cells led to increased proliferation and migration, and reduced apoptosis, with effects reversed by the FGFR inhibitor PD173074 (Yue et al., 2021; Mahmood et al., 2022). Furthermore, we created a suite of robust human cervical cell line (HCCCL) models (HeLa, SiHa, and CaSki) that are resistant to this drug (Bou Antoun et al., 2025). Here we leverage these HCCCLs to elucidate the molecular mechanisms underlying resistance to PD173074. By comparing the transcriptomic, biochemical and functional profiles of PD173074-resistant HCCCLs to their parental counterparts, we have unravelled signalling networks that help maintain the resistant phenotype, opening avenues towards the development of novel therapeutics.

## 2 Materials and methods

### 2.1 Cell culture

The HCCCLs (CaSki, SiHa, and HeLa) were originally purchased the cell lines from American Type Culture Collections (ATCC, Manassas, VA, United States). The corresponding PD173074 resistant derivatives, which have been previously characterized, were cultured under conditions described previously (Bou Antoun et al., 2025). HCCCLs were maintained in Dulbecco’s Modified Eagle’s Medium (DMEM) (D5796; Sigma-Aldrich, St. Louis, MO, United States) supplemented with 10% fetal bovine serum (FBS) (F9665; Sigma-Aldrich, St. Louis, MO, United States) and 100 μg/mL penicillin–streptomycin (P4333; Sigma-Aldrich, St. Louis, MO, United States) at 37 °C in a humidified atmosphere with 5% CO_2_. The DR cells were cultured without the drug for a week before performing each experiment.

### 2.2 FGFR stimulation and inhibition

FGFR stimulation and inhibition were carried out as previously described (Bou Antoun et al., 2025). Briefly, before treatment, 2 × 10^5^ cells were plated in 6-well plates (3516; Corning Costar treated plates, NY, United States) and serum starved overnight at 70%–80% confluence. Cells were then stimulated with 100 ng/mL FGF2 plus 300 ng/mL heparin for 15, 30, or 60 min. In parallel, cells were pre-treated for 1 h with either DMSO (0.01%) (D2650; Sigma-Aldrich, St. Louis, MO, United States) as a vehicle control or 2 µM PD173074 (P2499; Sigma-Aldrich, St. Louis, MO, United States) before ligand exposure.

In addition to PD173074, the mTORC1 inhibitor everolimus (TRC-E945400; Toronto Research Chemicals, North York, ON, Canada) and the FAK inhibitor GSK2256098 (HY100498; MedChemExpress, Monmouth Junction, NJ, United States) were used. In parallel experiments, before being exposed to 100 ng·mL^−1^ FGF2 ligand, cells were pre-treated overnight with DMSO (0.01%), 1 µM GSK2256098 or 10 nM everolimus. The cells were lysed for Western blotting at the appropriate times after treatment.

### 2.3 Transcriptomic analysis

In this experimental design, each of the three cell lines (the parental line and its respective drug-resistant derivatives) served as biological replicates for identifying commonly dysregulated genes, rather than generating technical triplicates for each line. This approach was chosen to prioritise the identification of conserved, biologically relevant resistance-associated changes across genetically distinct models, thereby enhancing the broader relevance of the findings. While this strategy may limit statistical power compared to within-line replication, it enables the identification of robust adaptive mechanisms shared across models, reflecting a pragmatic balance between biological breadth and practical considerations.

Six total RNA samples (one sample of each of the parental and their respective DR HCCCL) were sent to BGI Tech Solutions (Hong Kong) to be processed using the BGISEQ sequencing platform. Total RNA was purified from each of the parental and DR HCCCLs by using RNeasy Plus Mini Kit (74134; QIAGEN, Hilden, Germany) according to the manufacturer’s instructions. The Agilent 2100 Bioanalyzer (Agilent RNA 6000 Nano Kit) was used to measure the concentration and integrity of the RNA samples. RNA was fragmented followed by cDNA synthesis to construct the library (cDNA input was used for next-generation sequencing). Finally, the library was sequenced using BGISEQ-500 Platform using a Combinational Probe-Anchor Synthesis Sequencing Method and an improved technology of DNA Nanoballs (DNB). The original data were filtered to obtain Clean Data using the SOAPnuke v1.5.2 software. The hg19 reference genome was used to map the clean reads for statistical analysis. Hierarchical Indexing for Spliced Alignment of Transcripts (HISAT) software v2.0.4 (Kim et al., 2015) was used to align sequences.

After novel transcript detection using StringTie (Pertea et al., 2015) v1.0.4, (Trapnell et al., 2012), v2.2.1 (Cufflinks tools) and CPC (Kong et al., 2007) v0.9-r2 software, reference transcripts and novel coding transcripts were merged to get complete reference, then by using Bowtie2 v2.2.5 software, the clean reads were mapped to the complete reference (Langmead and Salzberg, 2012). RSEM v1.2.12 software was used to calculate the gene expression levels for each sample (Li and Dewey, 2011) which were calculated based on Fragments Per Kilobase of transcript per Million mapped reads (FPKM).

### 2.4 Bioinformatic analysis of transcriptomic data

Based on the gene expression levels, the DEGs were identified between samples using the PossionDis method. PossionDis, based on the Poisson distribution, was performed as previously described (Audic and Claverie, 1997), with fold Change ≥ 2.00 and false discovery rate (FDR) ≤ 0.001 set as the default threshold for defining significant differential expression. DEGs were classified according to official Gene Ontology (GO) classification, and GO functional enrichment was performed using Phyper, a function of R. The false discovery rate (FDR) was then calculated for each p value. In general, GO terms with an FDR not larger than 0.01 were defined as significantly enriched. DEGs were classified to pathways according to the official Kyoto Encyclopedia of Genes and Genomes (KEGG) pathway classification system (Kanehisa and Goto, 2000; Kanehisa et al., 2016; Kanehisa et al., 2025) and functional enrichment was performed using Phyper. Furthermore, the STRING v12 database (von Mering et al., 2005) was used to analyse the protein-protein interaction (PPI) and construct the interaction networks of DEGs. The predicted interactions between DEG-encoded proteins were obtained using homology-based methods with known proteins.

### 2.5 Quantitative polymerase chain reaction (QPCR)

The RNeasy Plus Mini Kit (74134; Qiagen, Hilden, Germany)) was used to extract total RNA then Superscript TM IV cell direct cDNA synthesis system kit (18091050 Invitrogen, Thermo Fisher Scientific Baltics UAB, Vilnius, Lithuania) was used to synthesise cDNA according to the manufacturer’s guidelines. Primer pairs (Table 1) were designed using NCBI BLAST for each DEG. Using a fast syber green master mix (4385612; applied biosystems, Thermo Fisher Scientific Baltics UAB, Vilnius, Lithuania), QPCR analysis was carried out in triplicate for each gene (primers, Table 1). *GAPDH* and *HPRT* were utilised as housekeeping genes for normalisation of expression, and a melting curve analysis was carried out following each reaction. Unless specified otherwise, all primers had an annealing temperature of 60 °C. Using a qPCR machine (PCRmax Eco 48 Real-Time PCR system; EcoStudy software v5.2.17, PCRmax Ltd., Stone, Staffordshire, United Kingdom), amplification cycle thresholds were established and the 2^−ΔΔCT^ method was employed for analysis (Livak and Schmittgen, 2001).

**TABLE 1:** QPCR primers designed for the DEGs.

Gene	Primer	Product length
*BMPER*	Forward GGTGCGCTGTGTTGTTCATT Reverse ATTGTGTCCTGCCTCCAGTG	167
*CCDN1*	Forward GCCGAGAAGCTGTGCATCTA Reverse ATGAACTTCACATCTGTGGCA	227
*DUSP4*	Forward GTCAACGTGCGCTGTAACAC Reverse TCATAGCCGCCTTTGAGCAG	251
*EPHA2*	Forward AAGGAAGTGGTACTGCTGGA Reverse TCTCCTCGGTACACCCAGTT	191
*IL11*	Forward TGAGCCTGTGGCCAGATACA Reverse CTGGGAATTTGTCCCTCAGC	162
*IL7R*	Forward AGCACAAAGCTGACACTCCT Reverse ATAGGATCCATCTCCCCTGAGC	170
*PHLDA1*	Forward ACCAAATACCGCACCCAC Reverse AGAAATGTGCTCGTCCCAC	147
*PLCB4*	Forward GCTGCACCGCCAACAAGAT Reverse CAGCACTGTCCTCTGTCACTT	156
*PLXNA2*	Forward AAAGCAACTGCCTCCCTCTG Reverse TGTCCTTGGCATAGAGCAGC	195
*TNC*	Forward TAACGGTGGTGGATTCTGGG Reverse CGGTTCGGCTTCTGTAACAAT	182
*TPBG*	Forward GAATTGGGGATTAAGCGGTC Reverse CTTCCCTCCAGAAAGTACGCA	152
*GAPDH*	Forward CAATGACCCCTTCATTGACC Reverse TTGATTTTGGAGGGATCTCG	249
*HPRT*	Forward GACCAGTCAACAGGGGACAT Reverse CCTGACCAAGGAAAGCAAAG	218

### 2.6 Western blot analysis

An equal number of cells for each treatment were lysed using Bolt™ LDS sample buffer (4×) (B0008; Novex, Life technologies, Carlsbad, CA, United States) mixed with 50 mM DTT (D9779; Sigma-Aldrich, St. Louis, MO, United States) and diluted to 2× with deionized water. Equal volumes of protein samples, containing 2 × 10^5^ cells each, were loaded in each lane and separated by electrophoresis on hand-cast 10% Tris gels. Proteins were transferred onto nitrocellulose membranes, membranes blocked with 5% bovine serum albumin (BSA) (A2153; Sigma-Aldrich, St. Louis, MO, United States) then incubated overnight at 4 °C with primary antibodies (Table 2) diluted in 5% BSA/phosphate buffered saline (PBS). The membranes were then incubated for 1 h at room temperature with secondary antibodies, IRDye® 680LT Donkey (926-68023; LI-COR, Lincoln, Nebraska, United States) and IRDye® 800CW Donkey (926-32212; LI-COR, Lincoln, Nebraska, United States), diluted 1:10,000. PBS (P4417; Sigma-Aldrich, St. Louis, MO, United States) containing 0.1% Tween 20 (PBST) was used to wash the membranes between antibodies three times 5 min each at room temperature. The Odyssey CLx infrared imaging system (LICOR-Biosciences, Lincoln, Nebraska, United States) was used to visualise bands on membranes using IMAGE STUDIO software (version 6.0, LI-COR Biosciences, Lincoln, Nebraska, United States) at 700 nm and 800 nm, which correspond to the red and green channels, respectively. The brightness and contrast were optimised to obtain a suitable signal-to-noise ratio. Blots were performed on separate membranes due to experimental constraints; comparisons were made using internal loading controls. Gels were run under identical conditions in the same electrophoresis tank, and membranes were imaged simultaneously.

**TABLE 2:** Primary antibodies used for Western blot (WB) and Immunocytochemistry (ICC) assays.

Primary antibodies	Clonality	Host/Isotype	Catalogue number	Company	Bands (KDa)	Working concentration
Akt	Polyclonal	Rabbit	9272 S	Cell signalling technology	60	1:1,000 (WB)
CD49 b (Integrin alpha 2) (SN0752)	Monoclonal	Rabbit	MA5-32306	Invitrogen	150	1:1,000 (WB) 1:200 (ICC)
FAK	Polyclonal	Rabbit	PA5-17591	Invitrogen	125	1:1,000 (WB) 1:100 (ICC)
HSC70	Polyclonal	Rabbit	PA5-24624	Invitrogen	70	1:1,000 (WB)
P44/42 MAPK	Monoclonal	Rabbit	4695 S	Cell signalling technology	42/44	1:1,000 (WB)
PHLDA1 [EPR6674]	Monoclonal	Rabbit	ab133654	Abcam	45	1:1,000 (WB) 1:200 (ICC)
Phospho-AKT (S473)	Polyclonal	Rabbit	9271 S	Cell signalling technology	60	1:1,000 (WB)
Phospho-akt (Thr308)	Polyclonal	Rabbit	9275 S	Cell signalling technology	60	1:1,000 (WB)
Phospho-FAK (Tyr397) (31H5L17)	Monoclonal	Rabbit	700,255	Invitrogen	125	1:1,000 (WB) 1:500 (ICC)
Phospho-p44/42 MAPK (T202/Y204)	Polyclonal	Rabbit	9,101	Cell signalling technology	42/44	1:1,000 (WB)
Phospho-S ribosomal protein (Ser235/236)	Polyclonal	Rabbit	2211 S	Cell signalling technology	32	1:1,000 (WB)
PLCB4	Polyclonal	Rabbit	PA5-67835	Invitrogen	120	1:500 (WB) 1:200 (ICC)
S6 ribosomal protein (5G10)	Monoclonal	Rabbit	2217 S	Cell signalling technology	32	1:1,000 (WB)

### 2.7 Immunocytochemistry

Cells (5 × 10^4^) were seeded in 24-well tissue culture plates (3524; Corning Costar treated plates, NY, United States) that contained 13 mm diameter glass coverslips. Once the cells reached 70%–80% confluency they were fixed with 10% formalin for 15 min at room temperature.

Fixed cells were permeabilized in 0.1% saponin/PBS for 10 min and blocked with 5% BSA in PBS for 45 min at room temperature. Cells were next incubated for 1 h with primary antibodies (Table2) diluted in 5% BSA/PBS. After washing three times with PBS, cells were incubated for 1 h with secondary antibody, fluorescein (F-2765; Invitrogen, Life Technologies Corporation, Eugene, OR, United States)) diluted 1:200 in 5% BSA/PBS, then washed three times with PBS and last washed with water before being mounted with Prolong™ Diamond Antifade with DAPI (Molecular Probes, P36971; Invitrogen, Life Technologies Corporation, Eugene, OR, United States). The EVOS microscope (Life Technologies, Carlsbad, CA, United States) was used to acquire fluorescent images at ×40 magnification. IMAGE J (developed by Wayne Rasband) was used to analyse fluorescence intensity (Schneider et al., 2012).

### 2.8 WST-1 proliferation assay after drug treatments

HCCCLs were seeded at 5 × 10^3^ cells per well in 96-well tissue culture plates (3596; Corning costar treated plates, NY, United States). The next day the cells were treated with 2 µM PD173074, 1 µM GSK2256098, 10 nM everolimus or DMSO (vehicle control) for 48 h and 72 h before WST-1 cell proliferation assay was performed according to the manufacturer’s instructions (05015944001; Roche Diagnostics GmbH, Mannheim, Germany). Cells were incubated with WST-1 reagent for 45 min at 37 °C, and each sample’s absorbance was measured at 420 nm using the infinite plate reader (Tecan, Infinite® 200 pro, Magellan™ software version 7.2 SP1, Männedorf, Switzerland).

### 2.9 Functional studies in transiently transfected cells expressing pgLAP1-PHLDA1-GFP

Cells were seeded at 5 × 10^3^ cells per well in 96-well tissue culture plates (3300; Corning Costar, NY, United States) for proliferation assay, and at 1 × 10^5^ cells per well in 6-well tissue culture plates (3516; Corning Costar treated plates, NY, United States) for transwell migration assays. When at 40%–60% confluency, cells were transiently transfected with pgLAP1 containing *PHLDA1*-GFP or inactive mutant *PHLDA1*-GFP constructs (Fearon et al., 2018), to overexpress *PHLDA1* gene using FuGENE transfection reagent (E2311; Promega, Madison, WI, United States) according to manufacturer’s instructions. The pgLAP1 containing *PHLDA1*-GFP or inactive mutant *PHLDA1*-GFP constructs were generated previously (Fearon et al., 2018; Clayton et al., 2023). The mutant *PHLDA1* construct was produced in which the amino acid residues 152–159 and 167–171, which correspond to the predicted locations for phosphatidyl-3,4,5-trisphosphate (PIP3) binding, were eliminated.

### 2.10 IncuCyte proliferation assay

As soon as the cells seeded in flat-bottomed 96-well tissue culture plates (3300; Corning costar, NY, United States) were transfected with the constructs, they were placed in the IncuCyte ZOOM system (Essen Bioscience, Ann Arbor, MI, United States) to monitor cell proliferation. After 48 h, the culture medium was replenished with fresh medium containing 10% serum, and cells were treated with either 2 μM PD173074 or DMSO (vehicle control) for 48 h. Images were captured every 3 h over a 96 h incubation period. Culture confluence over time was quantified using kinetic processing parameters based on time-lapse image acquisition. Graphs were generated by the IncuCyte software starting from the time of transfection.

For IC_50_ determination, cells were transfected with the same constructs and cultured in medium containing 10% serum. After 24 h, the medium was replenished, and cells were treated with increasing concentrations of PD173074 (0, 0.01, 0.1, 0.5, 1, 2, and 10 μM) or DMSO for 72 h. Following treatment, the plates were transferred to the IncuCyte system for live-cell imaging and analysis using the same imaging parameters described above. IC_50_ values were calculated using GraphPad Prism 10 (version 10.6.0; Dr. Harvey Motulsky, San Diego, CA, United States).

### 2.11 Apoptosis assays

As directed by the manufacturer, active caspase-3/7 was detected using CellEvent™ Caspase-3/7 detection reagents (C10431; Invitrogen, Life Technologies Corporation, Eugene, OR, United States) to assess the effect of overexpressing *PHLDA1* gene in all DR HCCCLs on apoptosis after being transfected for 48 h and treated with 2 μM PD173074 or DMSO (vehicle control) for 24 h.

### 2.12 Transwell cell migration assay

A Transwell cell migration assay was performed using BD Falcon cell culture 24-well plate Transwell inserts (353097; Falcon; 8 µm pore, Corning, Tewksbury, MA, United States) to measure cell migration. After coating the base of each Transwell chamber with 10 μg/mL fibronectin in serum-free medium for 1 h at 37 °C, the fibronectin solution was discarded, and the wells were blocked for 30 min with 0.1% BSA in serum-free medium at 37 °C. Next, 1 × 10^5^ cells suspended in 250 µL medium containing 2% serum were added to each insert and treated with either DMSO (vehicle control) or 2 µM PD173074, while medium containing 10% serum was added to the lower compartment. After 16 h, medium was removed from both the upper and lower compartments, and non-migrating cells were scraped off the insert’s upper surface using cotton buds. Cells that migrated were then trypsinised and quantified using a haematocytometer

### 2.13 Statistical analysis

GraphPad Prism 10 (version 10.6.0, Dr. Harvey Motulsky, San Diego, CA, United States) was used for statistical analysis. Each experiment was carried out at least three times, with separate independent biological replicates. Student’s t-test, one-way ANOVA, and two-way ANOVA were applied to the raw data as required, followed by Tukey’s *post hoc* multiple comparison tests.

## 3 Results

### 3.1 Three PD173074-resistant cervical cancer cell lines share fourteen common differentially expressed genes

**FIGURE 1:**
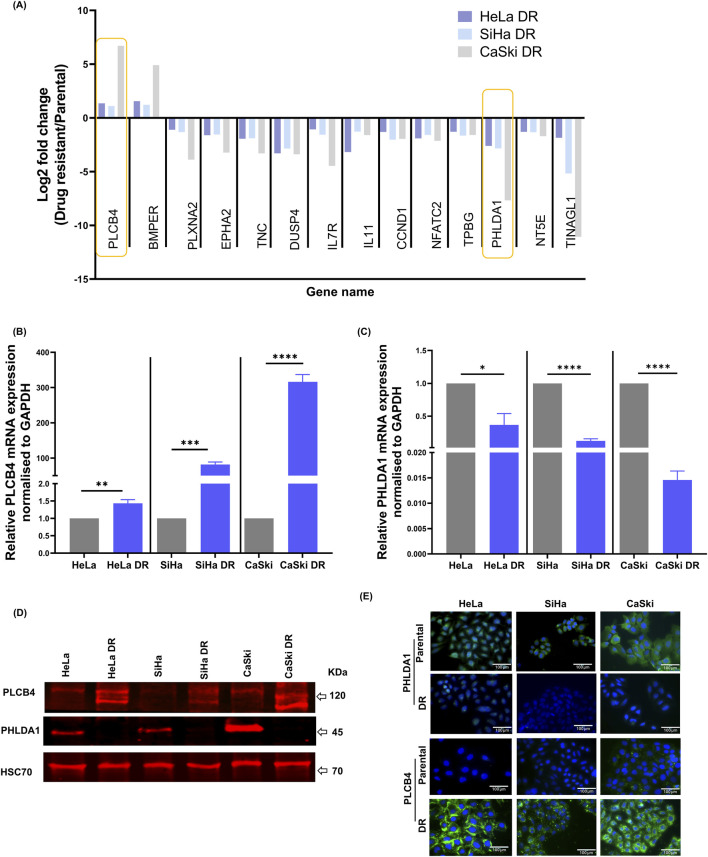
Transcriptomic analysis and validation of parental and PD173074-resistant human cervical cancer cell lines (HCCCLs). **(A)** Differentially expressed genes (DEGs; log2 fold change compared with parental values) in common between the three drug-resistant (DR) HCCCLs (HeLa DR, SiHa DR and CaSki DR) compared to their parental counterparts; positive values, upregulation; negative values, downregulation. **(B,C)** Relative mRNA expression (normalised to GAPDH and mean ± SEM, three independent experiments) of *PLCB4* and *PHLDA1,* respectively, in DR HCCCLs compared to their parental counterparts, which were assigned a relative value of 1. **(D,E)** Protein expression of PLCB4 and PHLDA1 in DR HCCCLs and their parental counterparts was validated using **(D)** Western blot (n = 3), and **(E)** immunocytochemistry (ICC) (n = 3). *p ≤ 0.05, **p ≤ 0.01, ***p ≤ 0.001, ****p ≤ 0.0001, compared to parental cell lines (t-test). All samples shown within each Western blot panel were run on the same gel and transferred to the same membrane. Comparisons are made between parental and DR cells under identical treatment conditions unless otherwise stated.

Transcriptomic analysis of three drug-resistant (DR) HCCCLs, and comparison to parental lines, identified differentially expressed genes (DEGs) common to all three cell lines, comprising two upregulated and 12 downregulated genes (Figure 1A). Among these, Phospholipase C beta 4 (*PLCB4)* and Pleckstrin Homology Like Domain Family A Member 1 (*PHLDA1)* were selected for further investigation, based on their potential relevance to drug resistance mechanisms in cancer (Li et al., 2017; Fearon et al., 2018; Wu et al., 2019; Zhang et al., 2019; Peng et al., 2022; Lee et al., 2023; Liu et al., 2025).

To validate the transcriptomic findings, we performed quantitative real-time-polymerase chain reaction (RT-PCR), Western blot and immunocytochemical analyses for all up-/downregulated genes. RT-PCR analysis confirmed significant upregulation of *PLCB4* and downregulation of *PHLDA1* mRNA in the DR HCCCLs compared to the parental controls (Figures 1B,C; Supplementary Figures S1, S2). Western blotting and immunocytochemistry corroborated these findings at the protein level, demonstrating an apparent increased expression of PLCB4 and decreased expression of PHLDA1 across all three DR HCCCLs (Figure 1D). PLCB4 was detected as a band at approximately 120–130 kDa, consistent with its expected molecular weight. Additional lower molecular weight bands observed in resistant cells may reflect isoforms, post-translational modification, degradation products. Importantly, the overall expression change between parental and resistant cells was consistent across experiments and aligned with the transcriptomic findings, supporting the robustness of the observed expression pattern. Furthermore, immunocytochemistry revealed enhanced cytoplasmic localization of PLCB4 and reduced cytoplasmic expression of PHLDA1 (Figure 1E). These data reveal for the first time that PLCB4 and PHLDA1 are consistently deregulated in DR HCCCLs, implicating them as novel mediators of chemoresistance.

### 3.2 Pathway enrichment and protein–protein interaction analysis of common differentially expressed genes in PD173074-resistant cervical cancer cell lines

To gain insight into the biological significance of the DEGs identified in all three DR HCCCLs, gene ontology (GO) (Supplementary Figure S3) and pathway enrichment analyses (Supplementary Figure S4) were performed on both the upregulated and downregulated gene sets identified in our transcriptome dataset (Supplementary Figures S3, S4).

**FIGURE 2:**
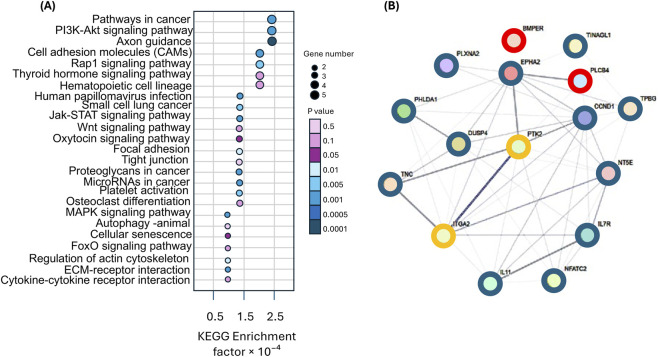
Pathway functional enrichment and protein-protein interaction (PPI) network of differentially expressed genes (DEGs). **(A)** Kyoto Encyclopedia of Genes and Genomes (KEGG) pathway enrichment analysis of DEGs shared across the three PD173074-resistant human cervical cancer cell lines (HCCCLs). The top 25 most enriched pathways of DEGs are presented. Circle size is proportional to the number of DEGs enriched in each pathway; circle colour corresponds to p-value. **(B)** STRING PPI network formed by the identified DEGs. Nodes encircled in red represent upregulated genes, those in blue indicate downregulated genes. Nodes in yellow are genes that interact strongly with the DEGs and serve as key connectors within the network. Edge thickness reflects interaction strength, with thicker lines denoting higher confidence or stronger predicted interactions. PTK2, protein tyrosine kinase 2 (5747) also known as focal adhesion kinase (FAK), phospholipase C beta 4 (PLCB4 (5332)), BMP-binding endothelial regulator (BMPER (168,667)), tenascin C (TNC (3371)), plexin A2 (PLXNA2 (5362)), Eph receptor A2 (EPHA2 (1969)), interleukin-7 receptor (IL7R (3575)), interleukin-11 (IL11 (3589)), cyclin D1 (CCND1 (595)), nuclear factor of activated T-cells, cytoplasmic 2 (NFATC2 (4773)), trophoblast glycoprotein (TPBG (7162)), dual specificity phosphatase 4 (DUSP4 (1846)), pleckstrin homology-like domain family A member 1 (PHLDA1 (22822)), ecto-5′-nucleotidase, also known as CD73 (NT5E (4907)) and tubulointerstitial nephritis antigen-like 1 (TINAGL1 (64129)), Integrin α2 (ITGA2 (3673). Numbers in brackets indicate the NCBI Gene (Entrez) ID for each gene.

GO analysis revealed that the DEGs were predominantly associated with biological processes related to cell proliferation, stress response, and signal transduction and significantly enriched in pathways involved in signalling as well as in cellular and metabolic processes (Supplementary Figures S3, S4). KEGG analysis further highlighted DEG involvement in pathways such as the PI3K-AKT and MAPK signalling, autophagy and adhesion processes, including focal adhesion (Figure 2A). Collectively these results provide important context to the transcriptomic data, suggesting that common mechanisms of drug resistance across the three HCCCL models may involve altered signalling, survival, and metabolic adaptation processes.

We next constructed and analysed the putative protein–protein interaction (PPI) network of the common DEGs using curated interaction databases, revealing potential interactions among the up- and downregulated proteins, suggesting coordinated functional roles in drug resistance (Figure 2B). Briefly, high-confidence interactions were highlighted, including ephrin type-A receptor 2 (EPHA2)–PLCB4 and interleukin-7 receptor (IL7R)–interleukin 11 (IL11) (Figure 2B). *EPHA2*, and cyclin D1 (*CCND1*) emerged as highly connected hub genes, participating in several interactions, suggesting their central roles in resistance-associated signalling networks. Most interactions involved proteins related to receptor signalling, cell cycle regulation, and immune modulation, highlighting potential mechanisms underlying the DR phenotype of HCCCLs.

### 3.3 *PHLDA1* overexpression restores phenotype in PD173074-resistant cervical cancer cell lines towards parental-like state

**FIGURE 3:**
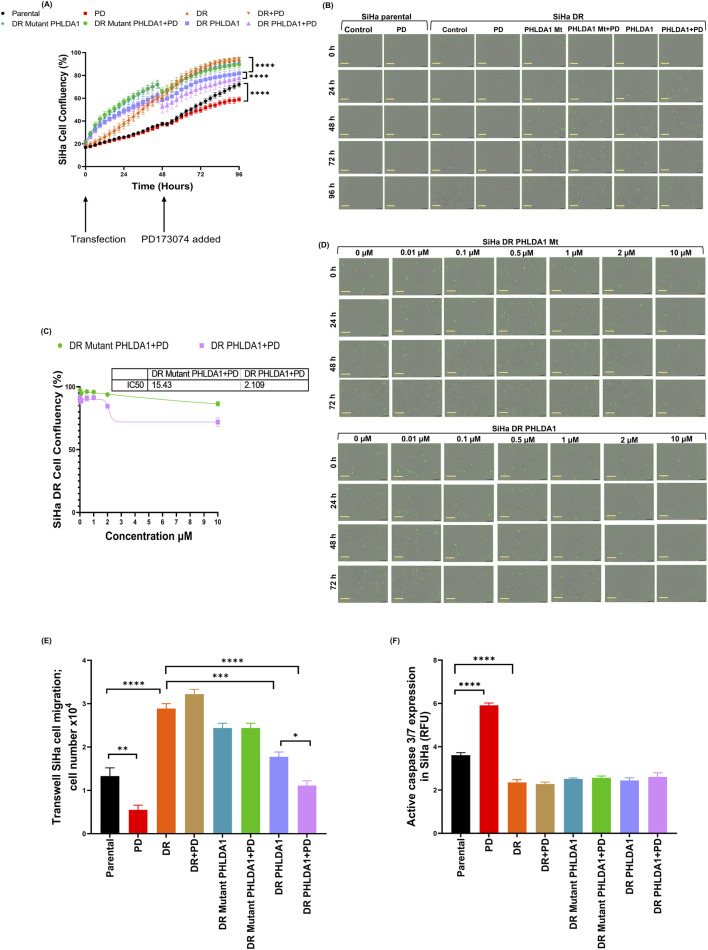
*PHLDA1* overexpression restores functional phenotype in PD173074-resistant human cervical cancer cell lines (HCCCLs) to parental-like state. **(A)** Cell proliferation of parental v’s drug resistant (DR), *PHLDA1* mutant (*PHLDA1 Mt*), and *PHLDA1* transfected SiHa DR cells treated with 2 μM PD173074 (PD) or DMSO (control). **(B)** Representative IncuCyte images (×10) showing proliferation of SiHa parental and DR cells with 2 μM PD173074 or DMSO; scale bar, 300 μm. **(C)** IC_50_ curves of *PHLDA1* transfected versus *PHLDA1 Mt* SiHa DR cells treated with PD173074 (0, 0.01, 0.1, 0.5, 1, 2 and 10 μM, 72 h). **(D)** Representative IncuCyte images (×10) showing proliferation of *PHLDA1 Mt* and *PHLDA1* transfected SiHa DR cells treated with (0–10 μM) PD173074 or DMSO; scale bar, 300 μm. **(E)** Transwell migration of indicated cell lines with2 μM PD173074 or DMSO after overnight incubation. **(F)** Caspase-3/7 activity (RFU) in parental vs. DR, *PHLDA1 Mt* and *PHLDA1* transfected SiHa DR cell lines with 2 μM PD173074 or DMSO for 24 h. The data represent the mean (±SEM) of three independent experiments. Differences between means were analysed with **(A)** two-way ANOVA or **(E,F)** one-way ANOVA, followed by Tukey’s *post hoc* test; *p ≤ 0.05, **p ≤ 0.01, ***p ≤ 0.001, ****p ≤ 0.0001, when compared with parental lines. Green fluorescence indicates GFP-tagged PHLDA1-expressing cells. Comparisons are made between parental and DR cells under identical treatment conditions unless otherwise stated.

Among the DEGs common to all three DR HCCCLs, we focused on *PHLDA1* because, in addition to its consistent downregulation, it has previously been identified as a key mediator of RTK-inhibitor resistance. In endometrial cancer, loss of PHLDA1 drives Akt-dependent compensatory signalling and promotes resistance to FGFR inhibition (Fearon et al., 2018). This prior evidence prompted us to investigate whether PHLDA1 plays a similar role in PD173074-mediated resistance in HCCCLs. We therefore overexpressed *PHLDA1* in each DR HCCCL model to evaluate whether restoring its expression would reverse the resistance phenotype. For clarity, we present here the results from the SiHa DR model (Figure 3); similar trends were observed across the two other HCCCLs, as detailed in the Supplementary Material (Supplementary Figures S5, S6).

Transient overexpression of *PHLDA1* in SiHa DR cells, followed by treatment with 2 μM PD173074 (PD), resulted in a significant reduction in both cell proliferation and migratory capacity compared to the control cells (Figures 3A,E). More specifically, live cell imaging (IncuCyte) analysis of cell confluency over 4 days revealed that DR SiHa cells exhibited greater proliferation than parental cells. Treatment with PD173074 substantially inhibited proliferation in parental cells but had minimal effect on DR cells (Figures 3A,B). However, overexpression of *PHLDA1* in DR cells significantly reduced proliferation compared to untreated DR controls, particularly when combined with PD173074. In contrast, DR cells transfected with a mutant PHLDA1 construct incapable of binding PIP3 (Fearon et al., 2018), showed no reduction in proliferation, regardless of PD173074 treatment, suggesting that PHLDA1 overexpression partially restores sensitivity to PD173074 by suppressing proliferative capacity in DR SiHa cells.

In addition, *PHLDA1*-overexpressing SiHa DR cells had a markedly lower IC_50_ to PD173074 compared to unmodified SiHa DR cells (Figures 3C,D). To ensure the specificity of these effects, we expressed the same non-functional mutant construct of *PHLDA1* as a control. The IC_50_ value of PD173074 decreased approximately 7.3-fold, from 15.4 μM in DR cells transfected with the mutant *PHLDA1* to 2.1 μM in DR cells transfected with the wild-type *PHLDA1* (Figure 3C), supporting a role for PHLDA1 in modulating the resistant phenotype.

Transwell cell migration assays (Figure 3E) revealed that the migratory capacity of DR SiHa cells was two-fold greater than that of parental cels. Whereas treatment with PD173074 alone had no effect on migration of DR cells, overexpression of *PHLDA1* led to a significant reduction in migration, particularly when combined with PD173074 treatment, restoring migratory behaviour towards to that of the parental cell line. In contrast, DR cells transfected with the mutant *PHLDA1* construct showed no significant change in migration, regardless of PD173074 treatment (Figure 3E; Supplementary Figures S5E, S6E). Furthermore, no significant differences in cell apoptosis were observed between *PHLDA1*-overexpressing SiHa DR and control cells (Figure 3F). More specifically, Caspase-3/7 activity assays (Figure 3F) revealed a marked increase in apoptosis in parental cells treated with PD173074, while DR cells exhibited significantly lower caspase-3/7 activity, consistent with a resistant phenotype. Notably, *PHLDA1* overexpression, with or without PD173074 treatment, did not significantly alter caspase activity in DR cells (Figure 3F; Supplementary Figures S5F, S6F), suggesting that the effect of *PHLDA1* on reversing drug resistance is independent of apoptosis induction. These findings indicate that *PHLDA1* overexpression restores aspects of PD173074 sensitivity in DR HCCCLs, reversing their proliferative and migratory advantages without engaging apoptotic pathways.

### 3.4 Phosphorylation-state profiling in PD173074-resistant cells suggests an AKT-independent signalling mechanism involving mTORC1

**FIGURE 4:**
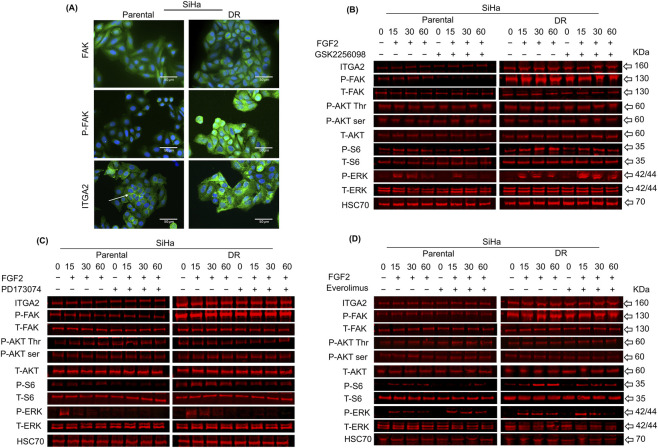
Multiple signalling pathways are differentially modulated in PD173074-resistant human cervical cancer cell lines (HCCCLs) compared to parental control cells **(A)** FAK (green) and ITGA2 (green) protein expression in parental and DR SiHa CCCLs revealed by immunocytochemistry (n = 3). Phosphorylated FAK (P-FAK, green) and ITGA2 expression were visibly greater in SiHa DR cell lines than parental with P-FAK predominately localized in the nucleus and cytoplasm and ITGA2 localised to the cytoplasm instead of the plasma membrane (parental cells). Nuclei were stained with DAPI (blue); scale bar, 50 μm. **(B–D)** FAK, AKT, ERK and S6 phosphorylation (p-FAK, p-AKT (ser and Thr308), p-ERK and p-S6; activation) after FGF2 stimulation in SiHa parental versus SiHa DR CCCLs (n = 3). The CCCLs were stimulated for 15, 30, and 60 min with either **(B)** 1 μM GSK2256098, **(C)** 2 μM PD173074, or **(D)** 10 nM everolimus with FGF2 ligand; DMSO served as controls Similar findings were seen in the other HCCCLs. T, total; P, phospho. All samples shown within each Western blot panel were run on the same gel and transferred to the same membrane. Samples were run on separate membranes due to experimental constraints; comparisons were based on normalisation to internal loading controls. Gels were run under identical conditions and membranes were imaged concurrently.

Having established a functional role for PHLDA1 in modulating drug resistance, we next sought to further interrogate the mechanisms underlying PD173074 resistance in HCCCLs. Since both the PI3K and FAK pathways were implicated, we evaluated the impact of FAK, FGFR and mTOR inhibition on the phosphorylation (activation) of downstream signalling proteins, particularly AKT, ERK and S6, upon FGF2 stimulation (Figure 4; Supplementary Figures S7, S8). This approach allowed us to (i) gain insights into how each pathway contributes to signal transduction, and (ii) identify potential vulnerabilities in DR cells. Given that consistent results were observed across all three DR HCCCL models, only the data from the SiHa model are presented for clarity; data for the other HCCCLs are provided as supplementary results files (Supplementary Figures S7, S8).

We first evaluated PI3K/AKT activation, as PHLDA1 is known to regulate AKT signalling (Chen et al., 2018; Fearon et al., 2018; Clayton et al., 2023). However, phosphorylation of AKT at Ser473 and Thr308 residues remained unaltered in both parental and DR SiHa cells following 2 µM PD173074 treatment and FGF2 stimulation, supporting that AKT activation is independent of FGFR signalling and is not likely a driver of resistance (Figure 4C). However, as these cells contain integrated HPV16, which can influence PI3K/AKT pathway activity, the observed lack of change in AKT phosphorylation should be interpreted with caution, as viral oncogene signalling may contribute to maintaining AKT activation independently of FGFR (Surviladze et al., 2013). We therefore examined other downstream targets and observed that phosphorylation of S6, a readout of mammalian target of rapamycin complex 1 (mTORC1) activity, was downregulated in parental SiHa cells upon PD173074 treatment, but remained elevated in SiHa DR cells, indicating persistent mTORC1 signalling despite FGFR inhibition (Figure 4C). Treatment with the mTORC1 inhibitor everolimus further confirmed this, as phospho-S6 levels were significantly reduced in parental cells but remained unchanged in DR cells (Figure 4D; Supplementary Figures S7D, S8D), suggesting that the sustained mTORC1 activity in the resistant phenotype is AKT-independent. Furthermore, FGF2 stimulation enhanced S6 phosphorylation, particularly in DR cells, suggesting that FGFR activation contributes to S6 activity in the resistant phenotype.

To further understand the resistance-associated signalling network, we interrogated extended STRING PPI networks of the DEGs and identified integrin α2 (ITGA2) as a putative central hub, that was found to interact with multiple DEGs (Figure 2B). We therefore examined changes in expression and localization of ITGA2 across all the parental and DR cell lines. Immunocytochemistry revealed increased cytoplasmic expression of ITGA2 in DR cells, whereas in parental cells ITGA2 was predominantly localized to the plasma membrane between adjacent cells (Figure 4A; Supplementary Figure S8A).

Given the established role of integrins in activating FAK (Mitra and Schlaepfer, 2006; Sulzmaier et al., 2014; Tan et al., 2023), we next evaluated levels of activated FAK. In SiHa-DR cells, elevated FAK phosphorylation was seen in both the cytoplasm and nucleus, a pattern observed across all DR HCCCLs (Figure 4A; Supplementary Figures S7A, S8A). To test whether integrin/FAK signalling contributes to resistance, both parental and SiHa DR cells were treated with the FAK inhibitor GSK2256098; strikingly, FAK phosphorylation was diminished in parental cells but remained prominent in SiHa DR cells (Figure 4B), supporting resistance to FAK inhibition. Furthermore, FGF2 stimulation did not enhance FAK phosphorylation, which remained constitutively elevated in DR cells. AKT phosphorylation was unaffected by either FGF2 stimulation or FGFR inhibition (Figure 4C; Supplementary Figures S7C, S8C), providing further support for an FGFR- and AKT-independent resistance mechanism. No significant changes were observed in other pathways post-FAK inhibition (Figures 4B; Supplementary Figures S7B, S8B). Interestingly, while FGFR inhibition significantly reduced S6 phosphorylation in parental cells, it had no effect on FAK phosphorylation in either parental or DR cells (Figure 4C; Supplementary Figures S7C, S8C).

Notably, DR cells also exhibited consistently elevated ITGA2 expression compared to parental lines, independent of FGF2 stimulation, which was seemingly unaffected by inhibition of FGFR, FAK or mTOR pathways (Figures 4B–D; Supplementary Figures S7B, S8B). Finally, following FGF2 stimulation, both parental and DR cells exhibited sustained ERK phosphorylation from 15 to 60 min. However, FGFR inhibition abolished ERK phosphorylation in both cell types, indicating effective FGFR pathway inhibition and confirming the well-established FGFR pathway dependence for ERK signalling (Figure 4C; Supplementary Figures S7C, S8C). Interestingly, however, FGF2 enhanced S6 phosphorylation, particularly in DR cells (Figures 4B–D; Supplementary Figures S8B–D). Yet, despite FGFR inhibition or mTORC1 blockade, S6 phosphorylation persisted in DR cells (Figure 4D; Supplementary Figures S7D, S8D). These findings indicate that PD173074 resistance in HCCCLs is driven by persistent FGFR-independent activation of integrin/FAK and mTORC1signalling, rather than AKT-mediated signalling.

### 3.5 Effect of combination treatments on proliferation of parental and PD173074-resistant cervical cancer cell lines

**FIGURE 5:**
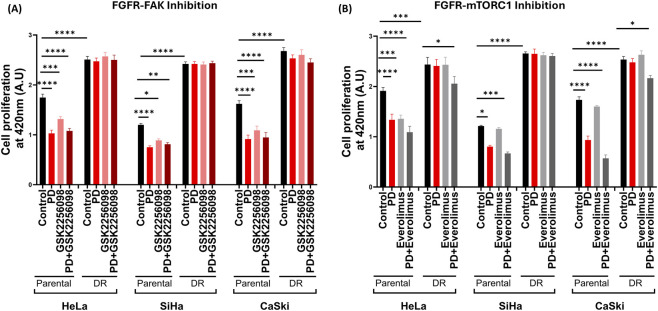
Dual FGFR–mTORC1 inhibition partially restores PD173074 sensitivity of drug resistant (DR) human cervical cancer cell lines (HCCCLs), whereas FGFR–FAK co-targeting was ineffective. The effect of 2 µM PD173074 (PD) with or without **(A)** 1 μM GSK2256098 (GSK), or **(B)** 10 nM everolimus, on cell proliferation in parental and DR HCCCLs; DMSO was used as control. Following a 72 h exposure to each drug combination, cell proliferation was measured using WST-1 reagent. Absorbance (arbitrary units (A.U.)) was measured at 420 nm. The data represent the mean (±SEM) of three independent experiments. Differences between means were analysed with two-way ANOVA followed by Tukey’s *post hoc* test; *p ≤ 0.05, **p ≤ 0.01, ***p ≤ 0.001, ****p ≤ 0.0001.

In all three parental cell lines, inhibition of either FGFR or FAK signalling significantly reduced proliferation (Figure 5A). However, combination treatment did not produce an additive effect. DR cells showed resistance to the single PD173074 or GSK2256098 treatments, and the combination of two treatments did not further suppress cell proliferation beyond that observed with single drug treatments or untreated cells (Figure 5A). Therefore, combination treatment did not restore sensitivity in DR cell lines.

Similar outcomes to those seen with dual FGFR and FAK inhibition were observed with combining PD173074 and everolimus (an mTORC1 inhibitor). In parental cells, everolimus alone only had a significant effect on HeLa cells. However, PD173074 alone and in combination with everolimus, had a significant inhibitory effect in all parental cell lines, with the combination treatment displaying even greater reduction in proliferation (Figure 5B). Furthermore, while HeLa DR and CaSki DR cells showed resistance to single treatments, combination treatment resulted in significant suppression of proliferation compared to either monotherapy. This finding indicates a potential combination-induced sensitivity in DR cells upon dual FGFR-mTORC1 pathway inhibition. SiHa DR cells, however, were not sensitive to either single treatment (PD173074 or everolimus) or their combination.

## 4 Discussion and conclusion

The FGFR signalling pathway plays a critical role in important cellular functions, and aberrant activation of the pathway contributes to tumour progression in many contexts, including for cervical cancer (Chioni and Grose, 2021; Bou Antoun and Chioni, 2023). Several FGFR tyrosine kinase inhibitors (erdatinib, pemigatinib, and futibatinib) have been approved for treating patients with urothelial cancer and cholangiocarcinoma (Katoh et al., 2024), but not cervical cancer. However, while some patients experience a positive response albeit of short duration, others show no benefit (Katoh et al., 2024). Treatment with small molecule inhibitors targeting FGFR(s) can lead to chemoresistance by inducing compensatory activation of alternative signalling pathways, such as PI3K/AKT or MAPK, which allow cancer cells to bypass FGFR dependency and maintain cell proliferation and survival (Datta et al., 2017; Zhou et al., 2020; Li I. et al., 2025). Here, using previously characterized DR HCCCLs as a model (Bou Antoun et al., 2025), we explored mechanisms associated with resistance to FGFR targeted therapies, focusing on adaptive signalling responses rather than a single dominant driver.

Initial transcriptomic analysis, validated at gene and protein level, revealed a panel of fourteen genes differentially expressed across all three DR HCCCLs, providing strong evidence for their association with the resistance phenotype. The transcriptomic approach was designed to identify conserved resistance-associated changes across multiple models rather than cell line–specific adaptations. Two genes were prioritised for further study: *PLCB4* (upregulated in DR HCCCLs) and *PHLDA1* (downregulated in DR HCCCLs). Overexpression of *PHLDA1* in DR HCCCLS partially modulated the DR phenotype, with observed reductions in migration and proliferation and increased responsiveness to FGFR inhibition. However, these effects, though statistically significant, were modest, suggesting that PHLDA1 restoration alone is insufficient to fully reverse resistance. This supports the interpretation of PHLDA1 as a modulator of the resistant phenotype rather than a sole driver. However, PHLDA1 may contribute to resistance through modulation of non-apoptotic processes such as proliferation and migration (Sakthianandeswaren et al., 2011; Moad et al., 2013). In contrast, the upregulation of PLCB4 suggests a potential role in driving adaptive signalling in resistant cells. As a phospholipase involved in the generation of second messengers, PLCB4 may act upstream of key oncogenic pathways, including PI3K/AKT/mTOR and MAPK signalling (Follo et al., 2015; Hamdi et al., 2025), thereby contributing to the maintenance of proliferative and survival signals in the drug-resistant state (Owusu Obeng et al., 2020; Wu et al., 2023). While our data do not directly establish this mechanism, they support a model in which PLCB4 participates in downstream signalling rewiring that promotes resistance.

These results suggest that PLCB4 might help mediate or mark drug resistance, while PHLDA1 loss is associated with the resistant phenotype and likely contributes as part of a broader adaptive signalling network (e.g., involving ITGA2 and mTOR signalling) rather than acting as a sole driver. PHLDA1 can act as a tumour suppressor or resistance modulator (Zhao et al., 2015; Chen et al., 2018; Fearon et al., 2018; Durbas, 2025), and its downregulation is likely a key event contributing to the resistance phenotype. While our re-expression experiments support a functional role for PHLDA1, further studies using complementary loss-of-function approaches in parental cells will be required to determine whether PHLDA1 loss is sufficient to induce resistance. In addition, although expression was monitored using GFP-tagged constructs, direct protein-level validation would further strengthen these findings.

Based on our novel findings, both PLCB4 and PHLDA1 could serve as biomarkers of acquired resistance to FGFR inhibitors or represent targets for the development of combination therapies. Most other modulated genes were downregulated in DR cells, which may reflect a broad transcriptional reprogramming in response to chronic FGFR inhibition (Clayton et al., 2023). A role for reduced PHLDA1 expression in mediating drug resistance to receptor tyrosine kinase (RTK) inhibitors has been identified in a previous study on breast and endometrial cancers, where downregulation led to increased AKT activity and drug resistance (Fearon et al., 2018). Some research has also linked PLCB4 upregulation to poor prognosis, relapse and drug resistance in haematological and solid tumours. For example, PLCB4 expression was higher in paediatric acute myeloid leukemia (AML) patients who relapsed compared to those with long term remission (Wu et al., 2019). Similarly, PLCB4 expression has recently been shown to correlate with a poorer survival in patients with osteosarcoma (Liu et al., 2025). Moreover, drug sensitivity analyses revealed that high PLCB4 expression correlates with lower IC_50_ values for several chemotherapeutic agents, indicating increased drug sensitivity (Liu et al., 2025). PLCB4 has been reported to activate downstream signalling pathways such as PI3K/AKT and MAPK, providing a potential mechanism for cells to bypass FGFR dependency and maintain a metastatic profile. This has important therapeutic implications, as it suggests that targeting downstream effectors such as mTOR or MAPK/ERK may represent a strategy to overcome or delay resistance to FGFR-targeted therapies. Indeed, combination strategies targeting both receptor-level signalling and downstream pathways (including MEK and mTOR) have shown promise in overcoming adaptive resistance in preclinical models of RTK-driven cancers (Bockorny et al., 2018; Krook et al., 2020; Lau et al., 2021). In our drug resistant cervical cancer cell models, reduced EPHA2 expression was accompanied by increased PLCB4. This might reflect signalling reprogramming, whereby loss of Eph receptor input is compensated by upregulation of PLC-mediated pathways to sustain proliferative and pro-survival signalling. While direct evidence of simultaneous EPHA2 downregulation and PLCB4 upregulation in drug-resistant cervical cancer is lacking, analogous signalling rewiring phenomena have been reported in other RTK-driven resistance contexts (Song et al., 2020; Xiao et al., 2020; Yip and Papa, 2021; Tomuleasa et al., 2024). The pathway analysis of DEGs identified in DR HCCCLs suggests a broad reprogramming of cellular behaviours contributing to FGFR inhibitor resistance. For example, biological processes like (i) regulation of biological activity, (ii) response to stimulus and (iii) signal transduction are enhanced, suggesting DR HCCCLs have rewired signalling circuits and have adapted to stress.

Importantly, our study highlights rewiring of the integrin–FAK–mTOR axis as a potential mechanism underpinning therapeutic resistance to TKIs. Targeting ITGA2/FAK signalling can suppress cervical cancer growth and enhance chemotherapy response (Liu et al., 2016) and mTOR can be activated independently of AKT, contributing to drug resistance (Glaviano et al., 2023). Protein–protein interaction analysis highlighted ITGA2 as a regulatory hub protein. Upon engagement with the ECM, integrin clustering recruits and activates FAK at focal adhesion sites (Li et al., 2023). The observed overexpression of ITGA2 in DR cells may contribute to FAK activation, reinforcing adhesion-mediated survival and contributing to downstream signalling reprogramming involving an AKT independent mTOR pathway. Consistent with this, previous work in colon cancer cells demonstrated that PHLDA1 drives ITGA2 and ITGA6 expression and adhesion to collagen and laminin, while blocking these integrins phenocopied the effects of PHLDA1 loss (Sakthianandeswaren et al., 2011). This supports the view that PHLDA1 modulates adhesion and invasive behaviour through integrin-mediated signalling, reinforcing our ITGA2–FAK axis hypothesis as an important contributor to adaptive resistance. While increased ITGA2 expression and FAK activation suggest engagement of an alternative survival pathway, our data support involvement of this axis, rather than strict dependency. Importantly, in the absence of genetic perturbation or long-term dependency assays, conclusions regarding functional dependency cannot be drawn, and the ITGA2–FAK–mTOR axis should therefore be interpreted as a candidate component of adaptive signalling rather than a validated mechanistic driver of resistance.

Interestingly, while ERK signalling remained FGFR-dependent, DR HCCCLs showed persistent FAK and mTORC1 pathway activation in the absence of detectable AKT phosphorylation changes. Elevated ITGA2 expression and rewired FAK signalling appear to contribute to bypassing FGFR inhibition and may represent potential therapeutic vulnerabilities in resistant HCCCLs. DR cells consistently showed greater ITGA2 expression than their parental counterparts, regardless of FGF2 stimulation or inhibition of FGFR, FAK, or mTOR pathways, indicating that ITGA2 upregulation is a stable feature of the resistant phenotype rather than being dynamically regulated.

FGF2 treatment enhanced S6 phosphorylation, particularly in DR cells, implicating FGFR input into mTORC1 signalling in the resistant context. Indeed, even during FGFR inhibition or mTORC1 blockade, S6 phosphorylation persisted in DR cells, reinforcing the concept of the mTORC1 pathway decoupling from upstream regulators in resistance. The pathway functional enrichment data offer a snapshot of signalling networks altered in the DR HCCCLs, emphasising that resistance to FGFR inhibitors in cervical cancer is likely driven by system wide rewiring across oncogenic, adhesion, immune and metabolic pathways (Ahn et al., 2024; Zhang et al., 2024). For example, cancer related pathways were broadly affected (i.e., strong enrichment in Pathways in cancer, Proteoglycans in cancer and MicroRNAs in cancer). PI3K-AKT and MAPK signalling were also altered significantly, supporting our findings of persistent mTOR activity and ERK signalling despite upstream inhibition. The PPIs between the DEGs reveal a tight network linking oncogenic signalling (e.g., EPHA2, PLCB4), cell cycle (e.g., CCND1), adhesion and matrix remodelling (e.g., TNC, PLXNA2), and immune–inflammatory responses (e.g., IL7R, IL11, NT5E), further supporting the concept of pathway rewiring in FGFRi resistant HCCCLs.

Drug concentrations in this study were selected to achieve partial pathway inhibition, allowing the detection of adaptive signalling responses rather than maximal cytotoxic effects. Under these conditions, phenotypic changes were modest, reflecting the multifactorial nature of resistance. Consequently, IC_50_ values should be interpreted with caution, as responses did not reach full inhibitory ranges. This approach more closely reflects clinically relevant conditions, where incomplete pathway inhibition can promote adaptive resistance (Zhang et al., 2024; Isermann et al., 2025; Xin and Miao, 2026).

Functional studies showed that, while in parental CCCLs, inhibition of FGFR or FAK attenuated proliferation, additive or synergistic effects did not occur with combined treatment, suggesting functional overlap between these pathways. Pathway crosstalk can result in shared downstream signalling effects that might limit benefit from dual FGFR and FAK inhibition, consistent with previous findings that showed no additive effect when combining FGFR inhibitors with androgen receptor antagonists in prostate cancer cells, despite individual efficacy (Afshan et al., 2024). Similarly, FAK inhibition combined with cisplatin therapy did not enhance anti-proliferative effects in mesothelioma models, underpinning that when monotherapies are effective, they can fail to synergize (Laszlo et al., 2019). DR HCCCLs exhibited resistance to PD173074 and GSK2256098 as single agents, but combination treatment failed to restore sensitivity. This indicates that acquired resistance in our models involves signalling rewiring that bypasses kinase dependency for both FGFR and FAK and therefore targeting FGFR and FAK together is insufficient to elicit a meaningful therapeutic response. The lack of additive effect from combined FGFR and FAK inhibition may reflect functional and compensatory signalling by alternative RTKs (Singleton et al., 2013; Kim et al., 2018; Krook et al., 2020; Shan et al., 2024).

Interestingly, while parental cells responded to both single treatment with PD173074 and combination treatment with everolimus - with the combination producing a more pronounced anti-proliferative effect - DR cells showed a more heterogeneous response. HeLa and CaSki DR cells, which were resistant to either agent alone, but showed significantly reduced proliferation upon combination treatment. This suggests that simultaneous blockade of upstream FGFR signalling and downstream mTORC1 may be required to disrupt compensatory survival mechanisms in certain resistant cell types, supporting dual-pathway inhibition as a strategy to partially overcome acquired resistance in specific cellular contexts (Krook et al., 2020). However, SiHa DR cells did not respond to either single treatments or the combination, indicating a potentially different resistance mechanism that relies on alternative signalling axes (e.g., noncanonical FGFR signalling partners might include adhesion molecules and extracellular matrix proteins) (Ferguson et al., 2021). This highlights the heterogeneity of resistance across cervical cancer subtypes and underscores the need for further molecular profiling to tailor combination therapies effectively. Collectively, these findings highlight the heterogeneous and context-dependent nature of resistance mechanisms across models. Short-term proliferation assays (72 h) were used to capture early dynamic responses to treatment and signalling adaptation. While these assays are appropriate for detecting early changes, longer-term assays may be required to fully assess sustained effects on proliferation and survival.

Several limitations of this study should be acknowledged. First, the transcriptomic analysis was performed without within-cell-line biological replicates; therefore, the identification of differentially expressed genes should be interpreted with caution, as this design limits statistical power. Instead, the analysis was intended to identify conserved resistance-associated changes across multiple independent models. Second, while functional assays support a role for PHLDA1 loss and integrin–FAK signalling in resistance, causality has not been definitively established. Third, the absence of genetic perturbation experiments limits conclusions regarding pathway dependency. Future studies incorporating genetic perturbation approaches will be required to determine whether this signalling axis represents a functional dependency or a context-dependent adaptive response. Fourth, short-term assays may underestimate the contribution of these mechanisms to long-term survival. Despite these limitations, the consistency of findings across three independent models supports the robustness of the observed adaptive response.

**FIGURE 6:**
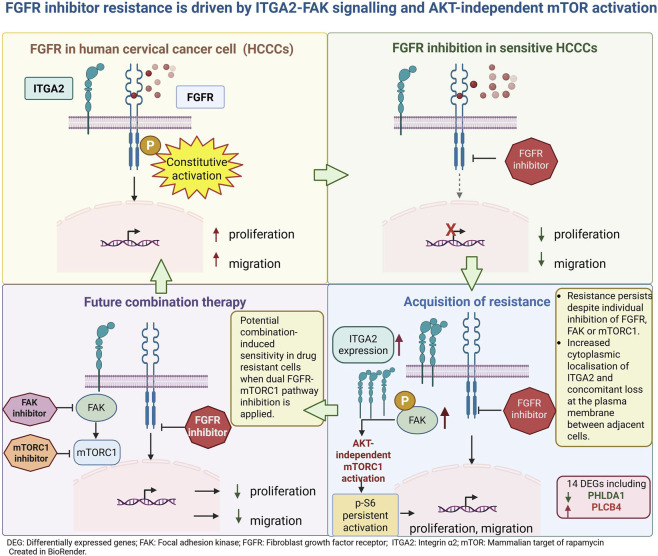
Proposed model of FGFR inhibitor resistance associated with ITGA2-FAK signalling and AKT- independent mTOR signalling. In FGFR-dependent human cervical cancer cells, FGFR inhibition suppresses proliferation and migration in sensitive cells but leads to acquired resistance via increased ITGA2 expression, FAK activation, and sustained mTORC1 signalling independent of AKT. Persistent p-S6 activation and transcriptional changes accompany resistance. Combined FGFR, FAK, or mTORC1 targeting is proposed as a strategy to overcome resistance. Created in BioRender.

In conclusion, by leveraging three complementary cell line models we provide novel insights into critical biological processes and molecular pathways commonly dysregulated in DR HCCCLs and suggest a network of interacting proteins that may contribute to the resistant phenotype. We show that ITGA2 and FAK signalling are associated with a compensatory signalling axis that may enable cells to adapt to FGFR blockade and sustain cell proliferation (Figure 6). Collectively, these findings point to resistance as a systems-level adaptation rather than an isolated molecular event, driven by widespread rewiring of oncogenic, adhesion, and metabolic networks. Recognising this complexity is essential for developing more effective strategies that target key signalling hubs rather than single pathways. By defining this integrated signalling architecture, our study provides a framework for future investigation of combination therapeutic strategies and identification of biomarkers predictive of therapeutic response in FGFR inhibitor–resistant cervical cancers.

## Data Availability

The raw sequence data reported in this paper have been deposited in the Genome Sequence Archive (Genomics, Proteomics & Bioinformatics 2025) in National Genomics Data Center (Nucleic Acids Res 2025), China National Center for Bioinformation / Beijing Institute of Genomics, Chinese Academy of Sciences (GSA-Human: HRA016082) (Members and Partners, 2025, Zhang et al., 2025) that are publicly accessible at https://ngdc.cncb.ac.cn/gsa-human/.
