# Supplementary material for: FGFR inhibitor resistance in cervical cancer: a role for integrin α2 and mTOR signalling
Source: Front Cell Dev Biol. 2026 Jun 4;14:1863679. doi: 10.3389/fcell.2026.1863679 (PMC13276803; doi:10.3389/fcell.2026.1863679)
Supplement: Supplementary file 1 [file DataSheet1.pdf]

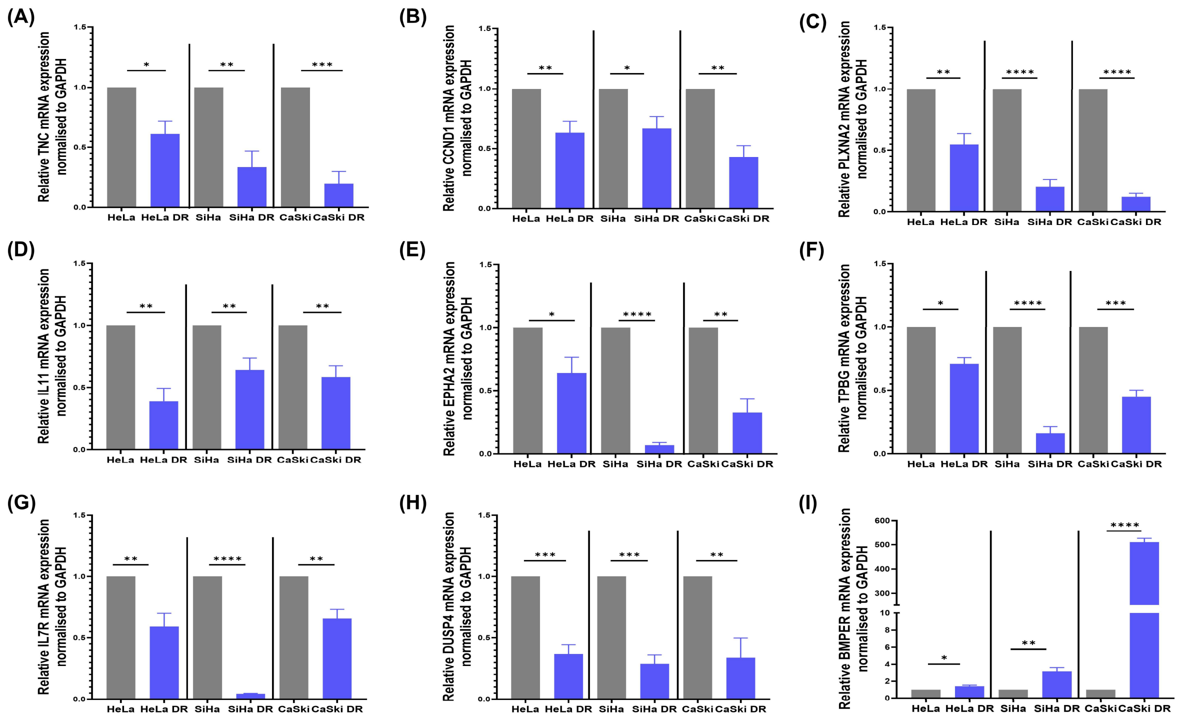

Figure S1. Relative mRNA expression of parental and PD173074-resistant human cervical cancer cell lines (HCCCLs) normalised to GAPDH. Relative mRNA expression of (A) TNC, (B) CCCND1, (C) PLXNA2, (D) IL11, (E) EPHA2, (F) TPBG, (G) IL7R, (H) DUSP4 and (I) BMPER in DR HCCCLs compared to their parental counterparts, which were assigned a relative value of 1. The data represent the mean (± SEM) of three independent experiments. Differences between means were analysed with t-test; * $p \leq 0.05$, ** $p \leq 0.01$, *** $p \leq 0.001$, **** $p \leq 0.0001$. Comparisons are made between parental and DR cells under identical treatment conditions.

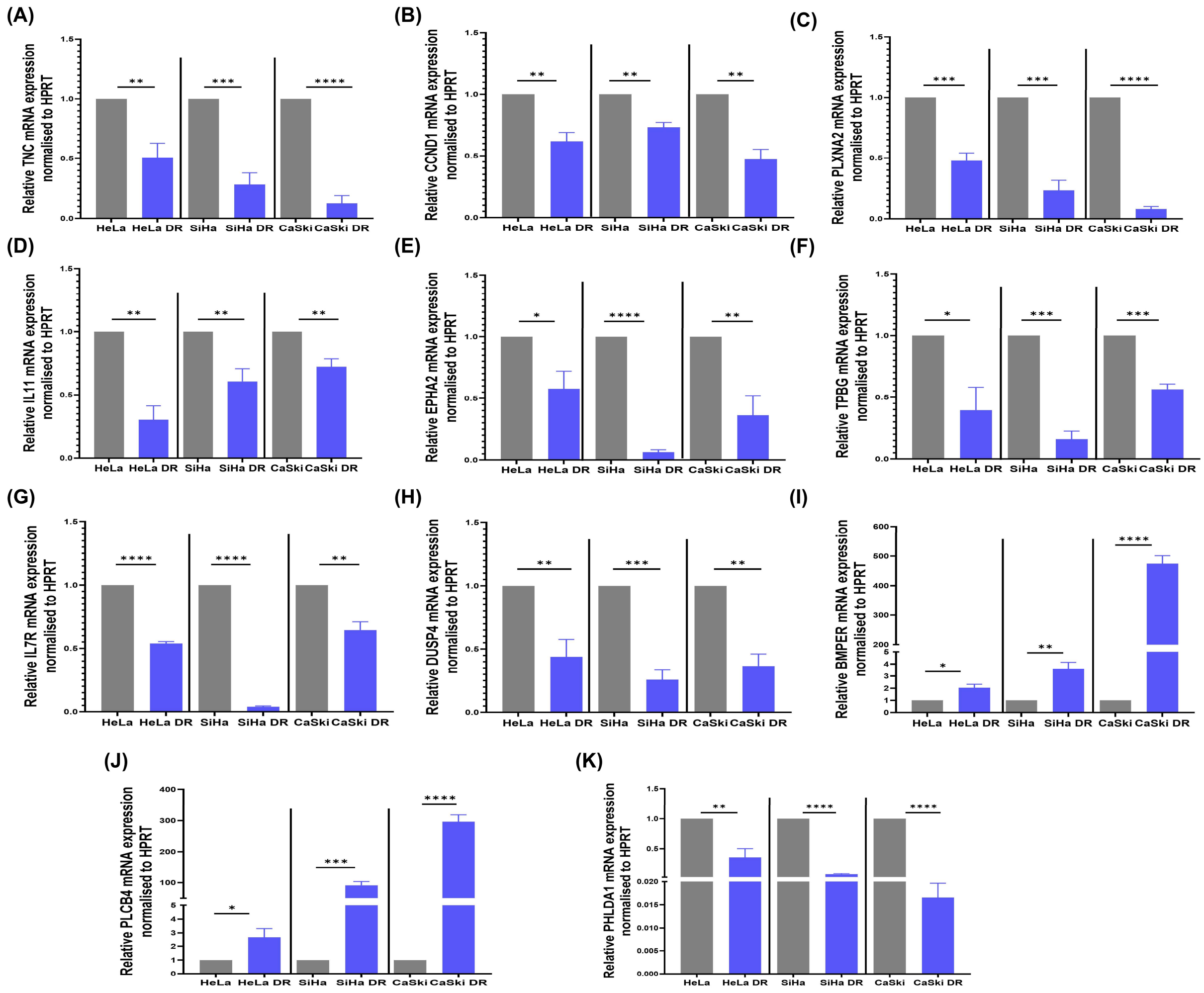

Figure S2. Relative mRNA expression of parental and PD173074-resistant human cervical cancer cell lines (HCCCLs) normalised to HPRT. Relative mRNA expression of (A) TNC, (B) CCCND1, (C) PLXNA2, (D) IL11, (E) EPHA2, (F) TPBG, (G) IL7R, (H) DUSP4 and (I) BMPER in DR HCCCLs compared to their parental counterparts, which were assigned a relative value of 1. The data represent the mean (± SEM) of three independent experiments. Differences between means were analysed with t-test; * $p \leq 0.05$, ** $p \leq 0.01$, *** $p \leq 0.001$, **** $p \leq 0.0001$. Comparisons are made between parental and DR cells under identical treatment conditions.

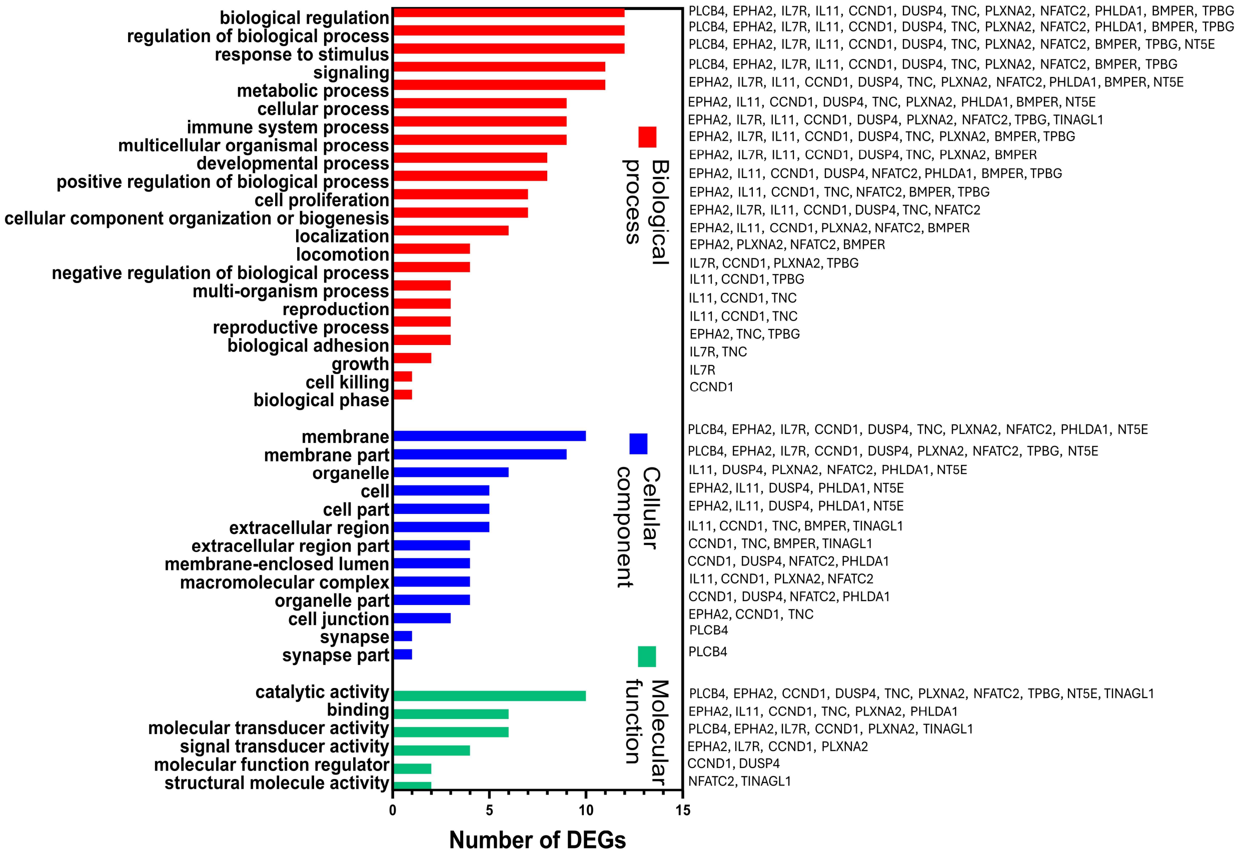

Figure S3. Gene Ontology (GO) analysis of DEGs. GO classification of DEGs that are in common between the three PD173074-resistant human cervical cancer cell lines (HCCCLs) where X axis represents number of DEGs and Y axis represents GO term. There are three categories of GO: biological process (red), cellular component (blue), and molecular function (green). On the right-hand side of the diagram, the names of the genes involved are listed for each GO term within each category. These gene names correspond to the DEGs that are enriched in the specific biological processes, molecular functions, or cellular components represented by the GO terms.

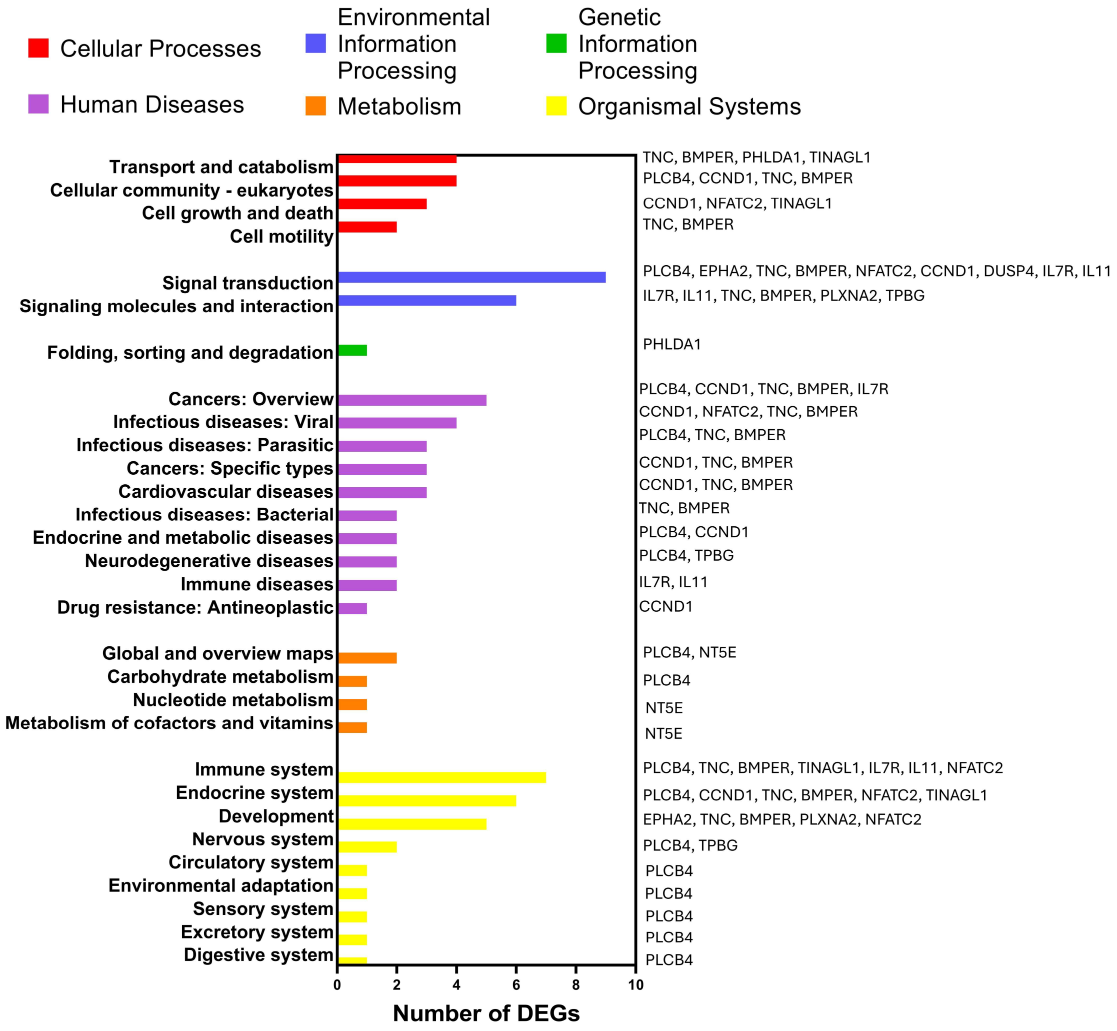

Figure S4. Pathway analysis of differentially expressed genes (DEGs). Kyoto Encyclopedia of Genes and Genomes (KEGG) Pathway classification of DEGs that are common to all three PD173074-resistant human cervical cancer cell lines (HCCCLs); X, number of DEGs; Y, functional classification of KEGG. The branches for KEGG pathways are: Cellular Processes (red), Environmental Information Processing (blue), Genetic Information Processing (green), Human Disease (purple), Metabolism (orange) and Organismal Systems (yellow). The names of genes involved in each pathway are listed on the right-hand side of the plot, providing a visual representation of the specific DEGs associated with the pathways.

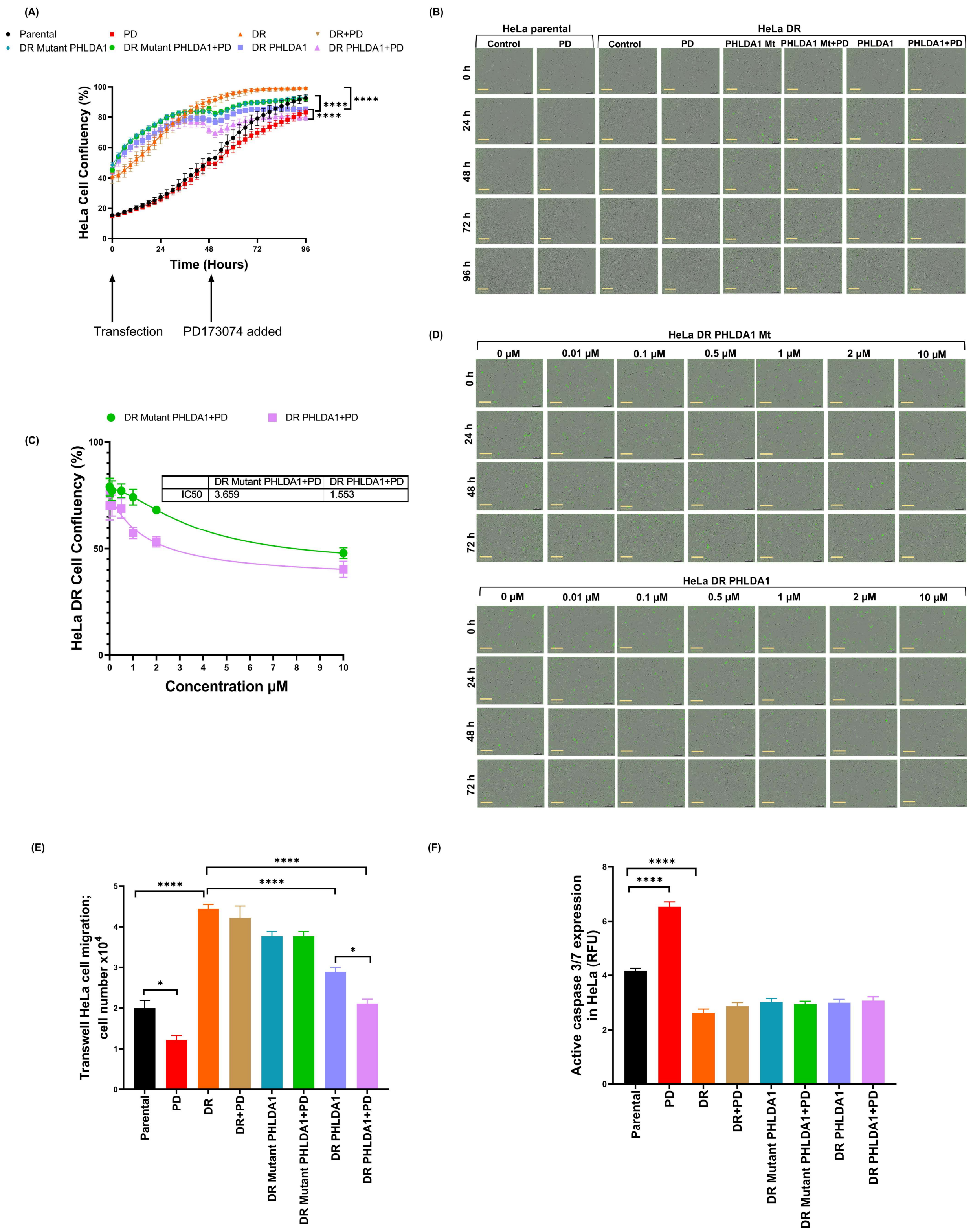
(A)
Parental
PD
DR
DR+PD
DR Mutant PHLDA1
DR Mutant PHLDA1+PD
DR PHLDA1
DR PHLDA1+PD
HeLa Cell Confluency (%)
Time (Hours)
****
****
****
Transfection
PD173074 added
(B)
HeLa parental
HeLa DR
Control
PD
Control
PD
PHLDA1 Mt
PHLDA1 Mt+PD
PHLDA1
PHLDA1+PD
0 h
24 h
48 h
72 h
96 h
(C)
DR Mutant PHLDA1+PD
DR PHLDA1+PD
HeLa DR Cell Confluency (%)
DR Mutant PHLDA1+PD
DR PHLDA1+PD
IC50
3.659
1.553
Concentration µM
(D)
HeLa DR PHLDA1 Mt
0 µM
0.01 µM
0.1 µM
0.5 µM
1 µM
2 µM
10 µM
0 h
24 h
48 h
72 h
HeLa DR PHLDA1
0 µM
0.01 µM
0.1 µM
0.5 µM
1 µM
2 µM
10 µM
0 h
24 h
48 h
72 h
(E)
Transwell HeLa cell migration; cell number x10^4
****
****
****
*
*
Parental
PD
DR
DR+PD
DR Mutant PHLDA1
DR Mutant PHLDA1+PD
DR PHLDA1
DR PHLDA1+PD
(F)
Active caspase 3/7 expression in HeLa (RFU)
****
****
Parental
PD
DR
DR+PD
DR Mutant PHLDA1
DR Mutant PHLDA1+PD
DR PHLDA1
DR PHLDA1+PD

Figure S5. *PHLDA1* overexpression restores functional phenotype in PD173074-resistant HeLa human cervical cancer cell lines (HCCCLs) to parental-like state. (A) Cell proliferation of parental v's drug resistant (DR), *PHLDA1* mutant (*PHLDA1 Mt*), and *PHLDA1* transfected HeLa DR cells treated with 2 μM PD173074 (PD) or DMSO (control). (B) Representative IncuCyte images (×10) showing proliferation of HeLa parental and DR cells with 2 μM PD173074 or DMSO; scale bar, 300 μm. (C) IC_{50} curves of *PHLDA1* transfected versus *PHLDA1 Mt* HeLa DR cells treated with PD173074 (0, 0.01, 0.1, 0.5, 1, 2 and 10 μM, 72 h). (D) Representative IncuCyte images (×10) showing proliferation of *PHLDA1 Mt* and *PHLDA1* transfected HeLa DR cells treated with (0 -10 μM) PD173074 or DMSO; scale bar, 300 μm. (E) Transwell migration of indicated cell lines with 2 μM PD173074 or DMSO after overnight incubation. (F) Caspase-3/7 activity (RFU) in parental v's DR, *PHLDA1 Mt* and *PHLDA1* transfected HeLa DR cell lines with 2 μM PD173074 or DMSO for 24 h. The data represent the mean (± SEM) of three independent experiments. Differences between means were analysed with (A) two-way ANOVA or (E, F) one-way ANOVA, followed by Tukey's post-hoc test; * $p \leq 0.05$, ** $p \leq 0.01$, *** $p \leq 0.001$, **** $p \leq 0.0001$, when compared with parental lines. Green fluorescence indicates GFP-tagged PHLDA1-expressing cells. Comparisons are made between parental and DR cells under identical treatment conditions unless otherwise indicated.

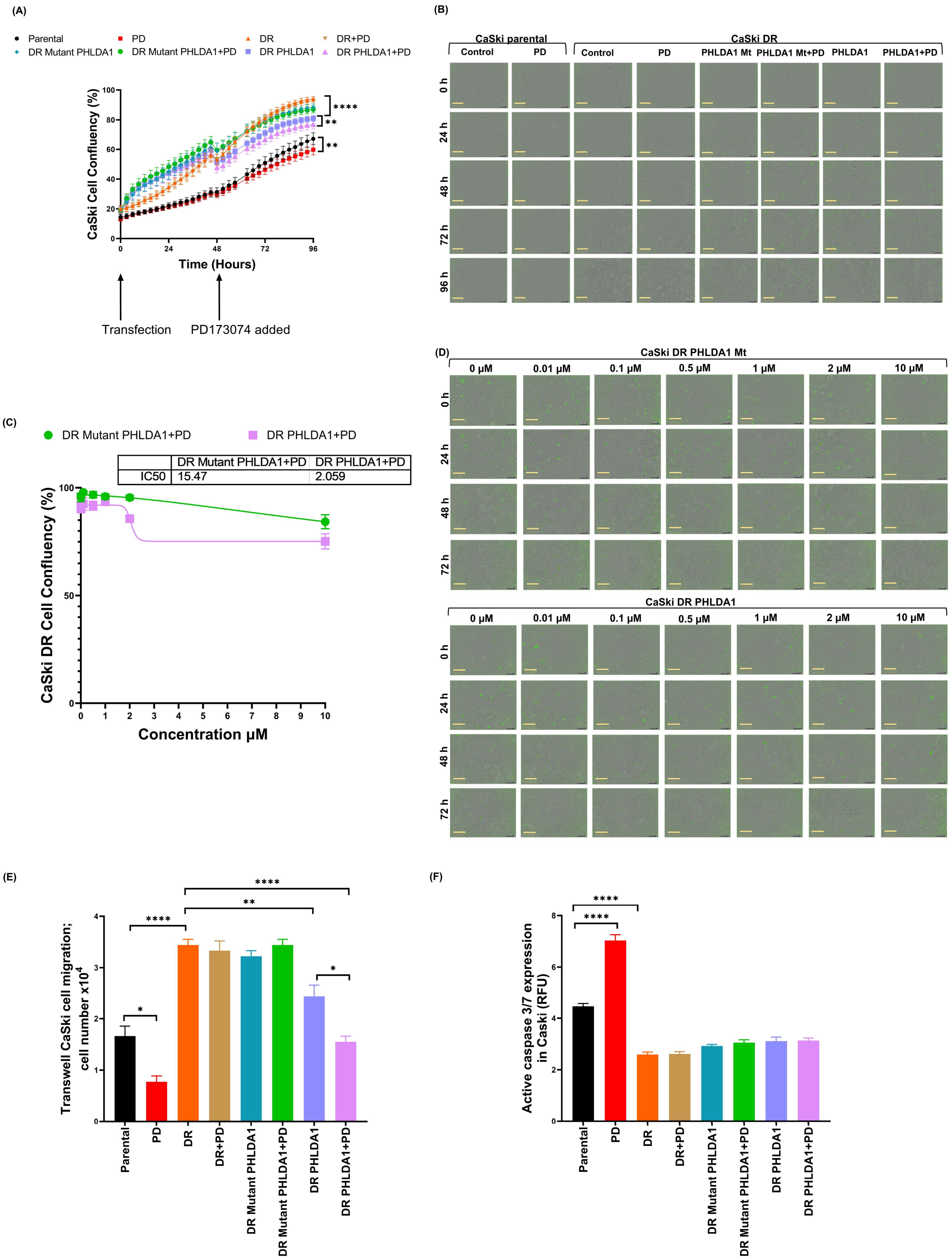
(A)
Parental
PD
DR
DR+PD
DR Mutant PHLDA1
DR Mutant PHLDA1+PD
DR PHLDA1
DR PHLDA1+PD
CaSki Cell Confluency (%)
Time (Hours)
****
**
**
Transfection
PD173074 added
(B)
CaSki parental
CaSki DR
Control
PD
PHLDA1 Mt
PHLDA1 Mt+PD
PHLDA1
PHLDA1+PD
0 h
24 h
48 h
72 h
96 h
(C)
DR Mutant PHLDA1+PD
DR PHLDA1+PD
IC50
15.47
2.059
CaSki DR Cell Confluency (%)
Concentration μM
(D)
CaSki DR PHLDA1 Mt
CaSki DR PHLDA1
0 μM
0.01 μM
0.1 μM
0.5 μM
1 μM
2 μM
10 μM
(E)
Transwell CaSki cell migration; cell number x10^4
Parental
DR Mutant PHLDA1
DR Mutant PHLDA1+PD
DR PHLDA1
DR PHLDA1+PD
(F)
Active caspase 3/7 expression in Caski (RFU)

Figure S6. *PHLDA1* overexpression restores functional phenotype in PD173074-resistant CaSki human cervical cancer cell lines (HCCCLs) to parental-like state. (A) Cell proliferation of parental v's drug resistant (DR), *PHLDA1* mutant (*PHLDA1 Mt*), and *PHLDA1* transfected CaSki DR cells treated with 2 μM PD173074 (PD) or DMSO (control). (B) Representative IncuCyte images (×10) showing proliferation of CaSki parental and DR cells with 2 μM PD173074 or DMSO; scale bar, 300 μm. (C) IC_{50} curves of *PHLDA1* transfected versus *PHLDA1 Mt* CaSki DR cells treated with PD173074 (0, 0.01, 0.1, 0.5, 1, 2 and 10 μM, 72 h). (D) Representative IncuCyte images (×10) showing proliferation of *PHLDA1 Mt* and *PHLDA1* transfected CaSki DR cells treated with (0 -10 μM) PD173074 or DMSO; scale bar, 300 μm. (E) Transwell migration of indicated cell lines with 2 μM PD173074 or DMSO after overnight incubation. (F) Caspase-3/7 activity (RFU) in parental v's DR, *PHLDA1 Mt* and *PHLDA1* transfected CaSki DR cell lines with 2 μM PD173074 or DMSO for 24 h. The data represent the mean (± SEM) of three independent experiments. Differences between means were analysed with (A) two-way ANOVA or (E, F) one-way ANOVA, followed by Tukey's post-hoc test; * $p \leq 0.05$, ** $p \leq 0.01$, *** $p \leq 0.001$, **** $p \leq 0.0001$, when compared with parental lines. Green fluorescence indicates GFP-tagged PHLDA1-expressing cells. Comparisons are made between parental and DR cells under identical treatment conditions unless otherwise indicated.

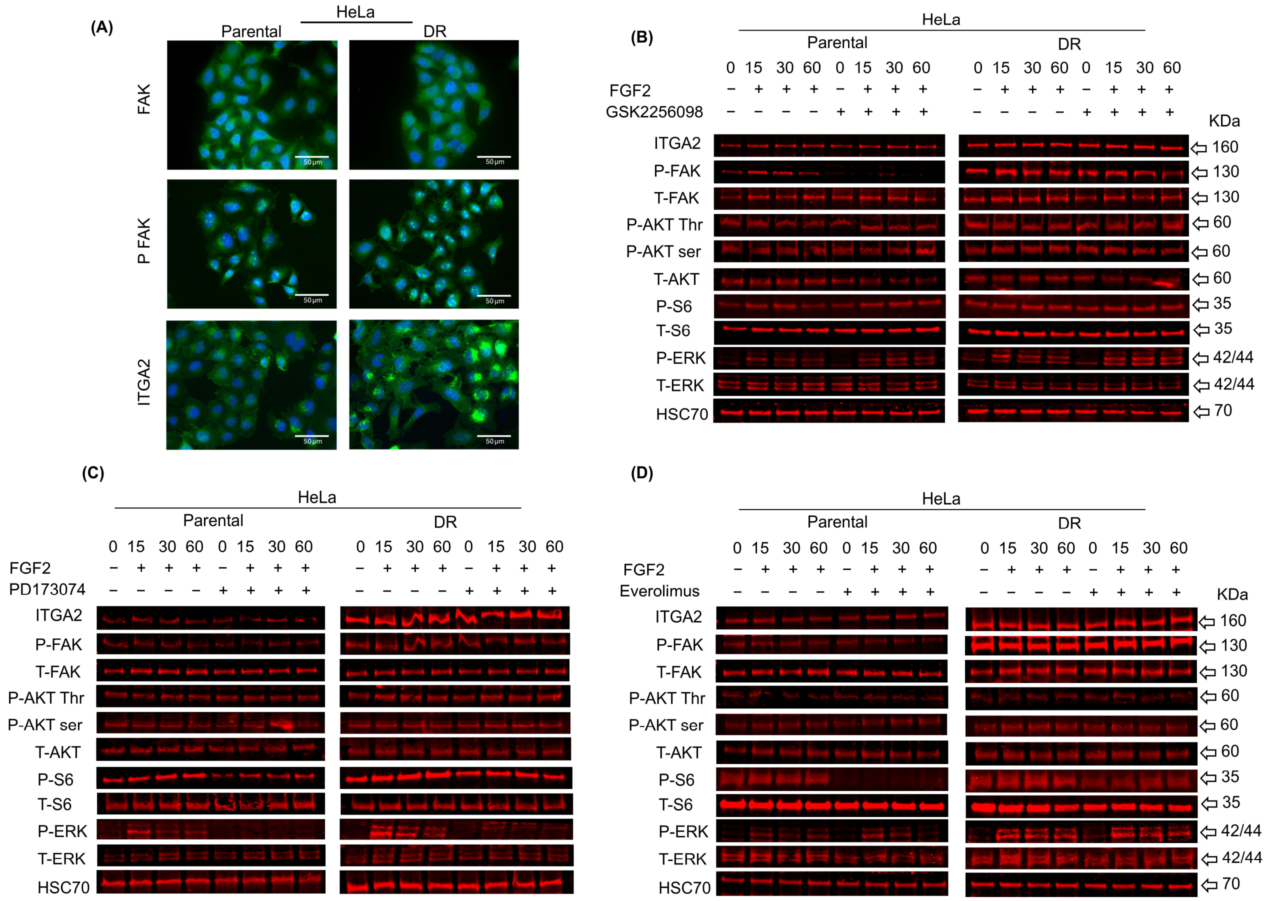

Figure S7. Multiple signalling pathways are differentially modulated in HeLa drug resistant (DR) human cervical cancer cell lines (HCCCLs) compared to parental control cells. (A) FAK (green) and ITGA2 (green) protein expression in parental and DR HeLa CCCLs revealed by immunocytochemistry (n=3). Phosphorylated FAK (P-FAK, green) and ITGA2 expression were visibly greater in HeLa DR cell lines than parental with P-FAK predominately localised in the nucleus and cytoplasm and ITGA2 localised to the cytoplasm and perinuclear instead of the cytoplasm (parental cells). Nuclei were stained with DAPI (blue); scale bar, 50 µm. (B-D) FAK, AKT, ERK and S6 phosphorylation (p-FAK, p-AKT (ser and Thr308), p-ERK and p-S6; activation) after FGF2 stimulation in HeLa parental versus HeLa DR CCCLs (n=3). The CCCLs were stimulated for 15, 30, and 60 min with either (B) 1 µM GSK2256098, (C) 2 µM PD173074 or (D) 10 nM everolimus with FGF2 ligand; DMSO served as controls. T: total; P: phospho. All samples shown within each western blot panel were run on the same gel and transferred to the same membrane. Samples were run on separate membranes due to experimental constraints; comparisons were based on normalisation to internal loading controls. Gels were run under identical conditions and membranes were imaged concurrently. Comparisons are made between parental and DR cells under identical treatment conditions unless otherwise indicated.

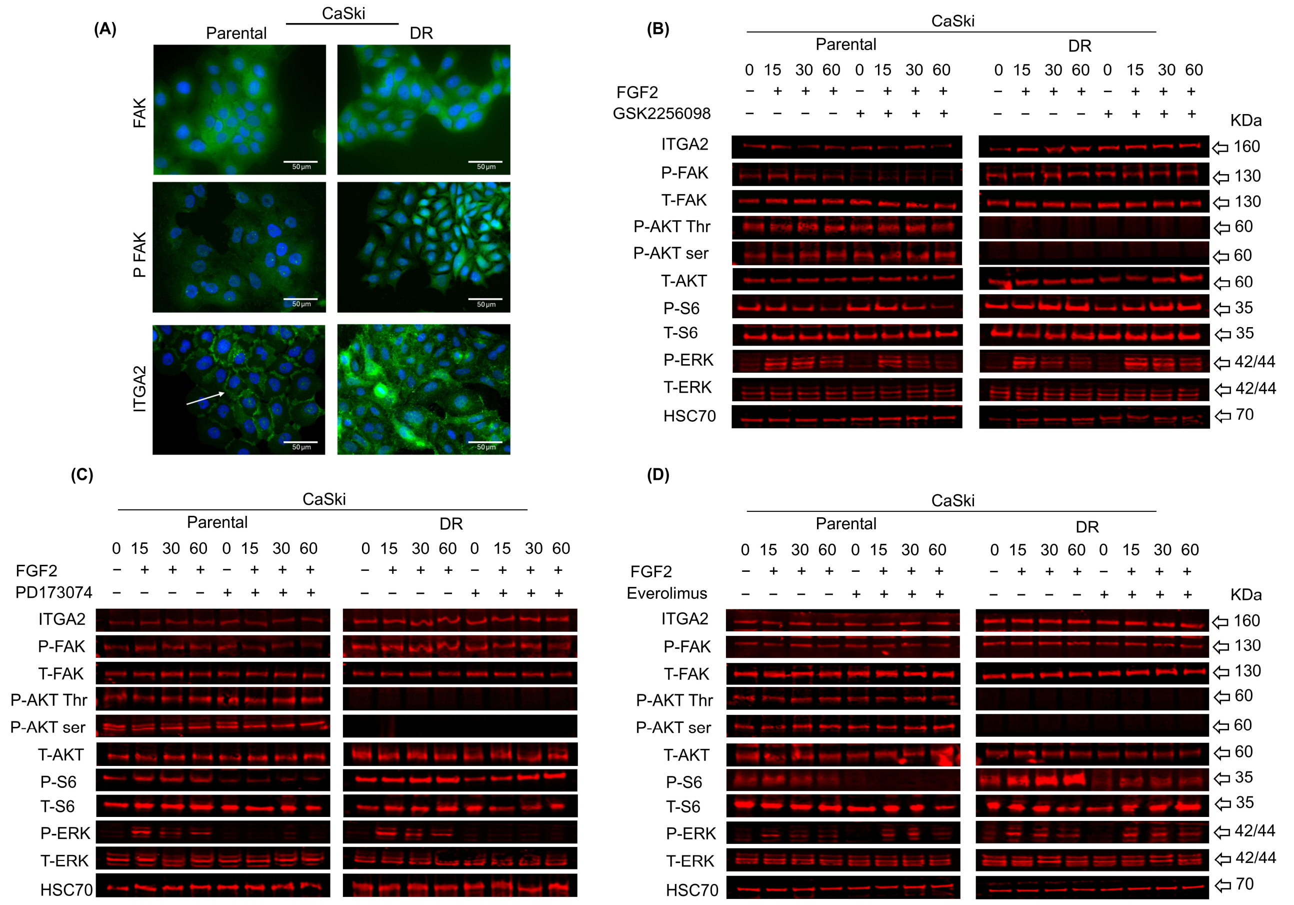

Figure S8. Multiple signalling pathways are differentially modulated in CaSki drug resistant (DR) human cervical cancer cell lines (HCCCLs) compared to parental control cells. (A) FAK (green) and ITGA2 (green) protein expression in parental and DR HeLa CCCLs revealed by immunocytochemistry (n=3). Phosphorylated FAK (P-FAK, green) and ITGA2 expression were visibly greater in CaSki DR cell lines than parental with P-FAK predominately localised in the nucleus and cytoplasm and ITGA2 localised to the cytoplasm instead of the plasma membrane (parental cells). Nuclei were stained with DAPI (blue); scale bar, 50 µm. (B-D) FAK, AKT, ERK and S6 phosphorylation (p-FAK, p-AKT (ser and Thr308), p-ERK and p-S6; activation) after FGF2 stimulation in CaSki parental versus CaSki DR CCCLs (n=3). The CCCLs were stimulated for 15, 30, and 60 min with either (B) 1 µM GSK2256098, (C) 2 µM PD173074 or (D) 10 nM everolimus with FGF2 ligand; DMSO served as controls. T: total; P: phospho. All samples shown within each western blot panel were run on the same gel and transferred to the same membrane. Samples were run on separate membranes due to experimental constraints; comparisons were based on normalisation to internal loading controls. Gels were run under identical conditions and membranes were imaged concurrently. Comparisons are made between parental and DR cells under identical treatment conditions unless otherwise indicated.
